# Distinct impacts of each anti-anti-sigma factor ortholog of the chlamydial Rsb partner switching mechanism on development in *Chlamydia trachomatis*

**DOI:** 10.1128/spectrum.01846-24

**Published:** 2024-10-29

**Authors:** Shiomi Junker, Vandana Singh, Aamal G. M. Al-Saadi, Nicholas A. Wood, Scott D. Hamilton-Brehm, Scot P. Ouellette, Derek J. Fisher

**Affiliations:** 1 Molecular Biology, Microbiology and Biochemistry Graduate Program, Southern Illinois University Carbondale, Carbondale, Illinois, USA; 2 Department of Pathology, Microbiology, and Immunology, College of Medicine, University of Nebraska Medical Center, Omaha, Nebraska, USA; 3 School of Biological Sciences, Southern Illinois University Carbondale, Carbondale, Illinois, USA; University of Guelph College of Biological Science, Guelph, Ontario, Canada

**Keywords:** RsbV, anti-anti-sigma factor, *Chlamydia*, development, phosphorylation

## Abstract

Partner switching mechanisms (PSMs) are signal transduction systems comprised of a sensor phosphatase (RsbU), an anti-sigma factor (RsbW, kinase), an anti-anti-sigma factor (RsbV, the RsbW substrate), and a target sigma factor. *Chlamydia* spp. are obligate intracellular bacterial pathogens of animals that undergo a developmental cycle transitioning between the infectious elementary body (EB) and replicative reticulate body (RB) within a host cell-derived vacuole (inclusion). Secondary differentiation events (RB to EB) are transcriptionally regulated, in part, by the housekeeping sigma factor (σ^66^) and two late-gene sigma factors (σ^54^ and σ^28^). Prior research supports that the PSM in *Chlamydia trachomatis* regulates availability of σ^66^. Pan-genome analysis revealed that PSM components are conserved across the phylum Chlamydiota, with *Chlamydia* spp. possessing an atypical arrangement of two anti-anti-sigma factors, RsbV1 and RsbV2. Bioinformatic analyses support RsbV2 as the homolog to the pan-genome-conserved RsbV with RsbV1 as an outlier. This, combined with *in vitro* data, indicates that RsbV1 and RsbV2 are structurally and biochemically distinct. Reduced levels or overexpression of RsbV1/RsbV2 did not significantly impact *C. trachomatis* growth or development. In contrast, overexpression of a non-phosphorylatable RsbV2 S55A mutant, but not overexpression of an RsbV1 S56A mutant, resulted in a 3 log reduction in infectious EB production without reduction in genomic DNA (total bacteria) or inclusion size, suggesting a block in secondary differentiation. The block was corroborated by reduced production of σ^54/28^-regulated late proteins and via transmission electron microscopy.

**IMPORTANCE:**

*Chlamydia trachomatis* is the leading cause of reportable bacterial sexually transmitted infections (STIs) and causes the eye infection trachoma, a neglected tropical disease. Broad-spectrum antibiotics used for treatment can lead to microbiome dysbiosis and increased antibiotic resistance development in other bacteria, and treatment failure for chlamydial STIs is a recognized clinical problem. Here, we show that disruption of a partner switching mechanism (PSM) significantly reduces infectious progeny production via blockage of reticulate body to elementary body differentiation. We also reveal a novel PSM expansion largely restricted to the species infecting animals, suggesting a role in pathogen evolution. Collectively, our results highlight the chlamydial PSM as a key regulator of development that could be a potential target for novel therapeutics.

## INTRODUCTION

The phylum Chlamydiota is comprised of obligate intracellular Gram-negative bacteria infecting a variety of eukaryotic hosts such as amoeba, invertebrates, and vertebrates including fish, reptiles, birds, and mammals (1). Like most obligate intracellular bacteria, they have reduced genomes varying in size from 1 Mbp to ~2.5 Mbp, and genome content impacts their endosymbiotic host range by dictating, for example, metabolic capacity/parasitic requirements, cell entry mechanisms, stress response pathways, and protein effector repertoires needed to survive in the face of different innate and adaptive immune responses (2–5). Despite variation in hosts and endosymbiotic requirements, all characterized members of the phylum undergo a developmental cycle involving differentiation of the infectious elementary body (EB) into the replicative reticulate body (RB) (primary differentiation) followed by replication of RBs and the eventual conversion of RBs into EBs (secondary differentiation) (6, 7). Halting developmental progression would be an effective means of preventing or treating infections, but the mechanisms governing and executing differentiation are poorly understood.

**Fig 1:**
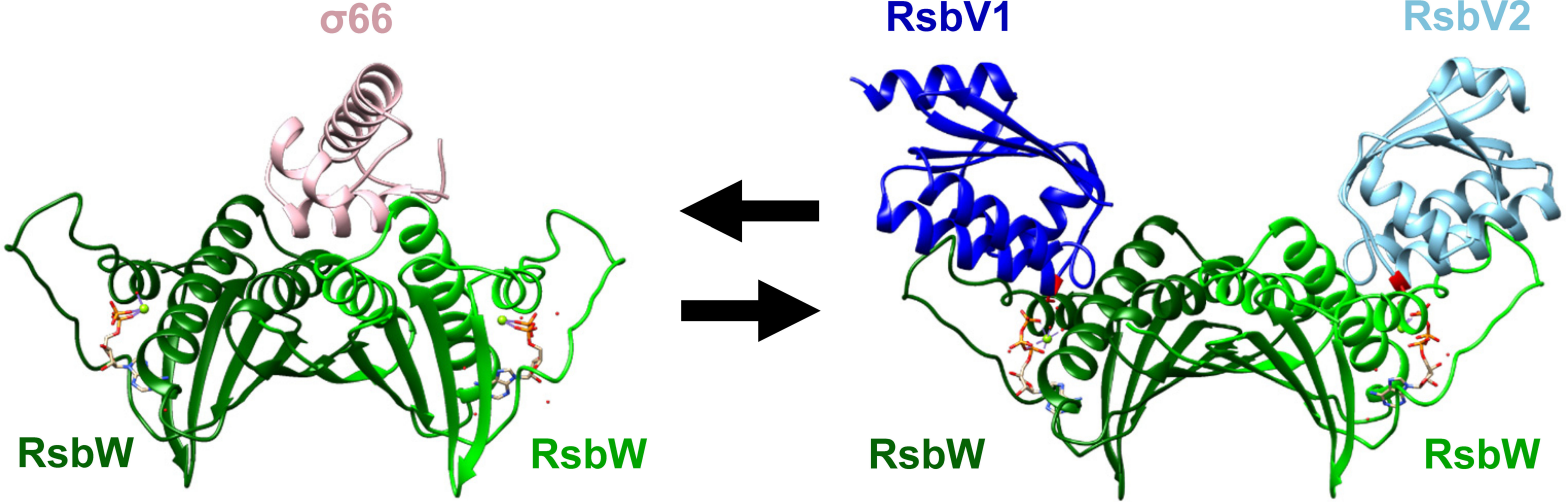
*In silico* structure models show RsbW switching protein interaction partners between σ^66^ and the AASFs RsbV1/RsbV2. Monomeric protein structures were predicted using AlphaFold. Each protein structure is superimposed with *Bacillus subtilis* homologs SpoIIAB-σ^B^ binding with ADP, Mg^2+^, H_2_O (1L0O) or SpoIIAB-SpoIIAA with ATP, Mg^2+^, H_2_O (1TID), and the structures were rendered using UCSF chimera. RsbW (dark green) is dimerized with another RsbW (light green), which interacts with a part of σ^66^ (pink) or AASFs (RsbV1 as dark blue and RsbV2 as sky blue). An ATP molecule is present in each RsbW monomer. The phosphorylation site of each AASF is highlighted in red (S56 for RsbV1, S55 for RsbV2).

Partner switching mechanisms (PSMs) are protein phosphorylation-based bacterial signal transduction systems that bacteria employ to sense and respond to environmental cues. Regulator of Sigma B, or Rsb, is a well-characterized PSM mostly found in Gram-positive bacteria where it is used to sequester the availability of σ^B^ in response to environmental or energy stressors (8–12). Release of the regulated sigma factor allows for transcriptional responses that can lead to developmental changes including spore formation, activation of virulence genes, motility, biofilm formation, or initiation of various stress response pathways (13–21). Nominally, a PSM is composed of an anti-sigma factor with kinase activity (ASF; RsbW), a sensor phosphatase (RsbU), and an anti-anti-sigma factor (AASF; RsbV) that is the substrate for both RsbW and RsbU (22, 23). The ASF switches between a complex with the sigma factor (transcription repressed) and the unphosphorylated AASF (sigma factor freed, transcription competent) (24–27) (see Fig. 1). Phosphorylation of the AASF by the ASF allows the ASF to switch back to the sigma factor (27–29). Activation of RsbU resets the AASF through dephosphorylation, allowing for complex formation with the ASF (30–32). In this way, transcription can be regulated in response to environmental cues or energy stress (33, 34).

The phylum Chlamydiota genomes contain a core PSM, which includes RsbU, RsbW (ASF), and RsbV (AASF) (5, 35). Studies of the PSM from *Chlamydia trachomatis* indicate that RsbU can sense the TCA-metabolite α-ketoglutarate (36) and glycolysis intermediate phosphoenolpyruvate (37), that RsbW can phosphorylate RsbV in an ATP-dependent manner (38–40), that RsbU can dephosphorylate RsbV (37–39), and that RsbW can complex with σ^66^ (housekeeping sigma factor, σ^70^ family member) (38). In *C. trachomatis* and other *Chlamydia* that infect vertebrates, there are two additional sigma factors, σ^54^ and σ^28^, that promote transcription of late genes involved in secondary differentiation (41–45). Transcription of *rpoD* (encoding σ^66^) is much higher than *rpoN* (encoding σ^54^) and *fliA* (encoding σ^28^) (44), and *in vitro* protein interaction assays with *Escherichia coli* sigma factors reveal that σ^70^ has the highest binding affinity with RNA polymerase (RNAP) followed by σ^54^, with σ^28^ having half the affinity of σ^54^ (46). The competition between sigma factors for the RNAP is important for the regulation of differentiation, and is driven by sigma factor abundance and RNAP affinity. Consequently, the ratio of σ^66^-RsbW to RsbW-RsbV in the bacterium impacts transcription by altering the pool of RNAP core available to σ^28^ and σ^54^ and would have a titration effect on σ^66^-dependent gene transcription with priority given to unrepressed consensus promoters when free σ^66^ levels are reduced. In this manner, the chlamydial PSM can potentially regulate growth rate and development in response to nutritional cues.

Here, we report on variations of the PSM across the phylum Chlamydiota and hypothesize that AASF expansion and alterations helped facilitate speciation of the animal-infecting *Chlamydia* (“pathogenic *Chlamydia*”). We also provide additional evidence supporting the hypothesis that the PSM functions in regulating secondary differentiation and that genetic manipulation of the PSM cripples production of EBs.

## RESULTS

### AASF gene copy number (RsbV versus RsbV1 and RsbV2) varies across the Chlamydiota phylum

**Fig 2:**
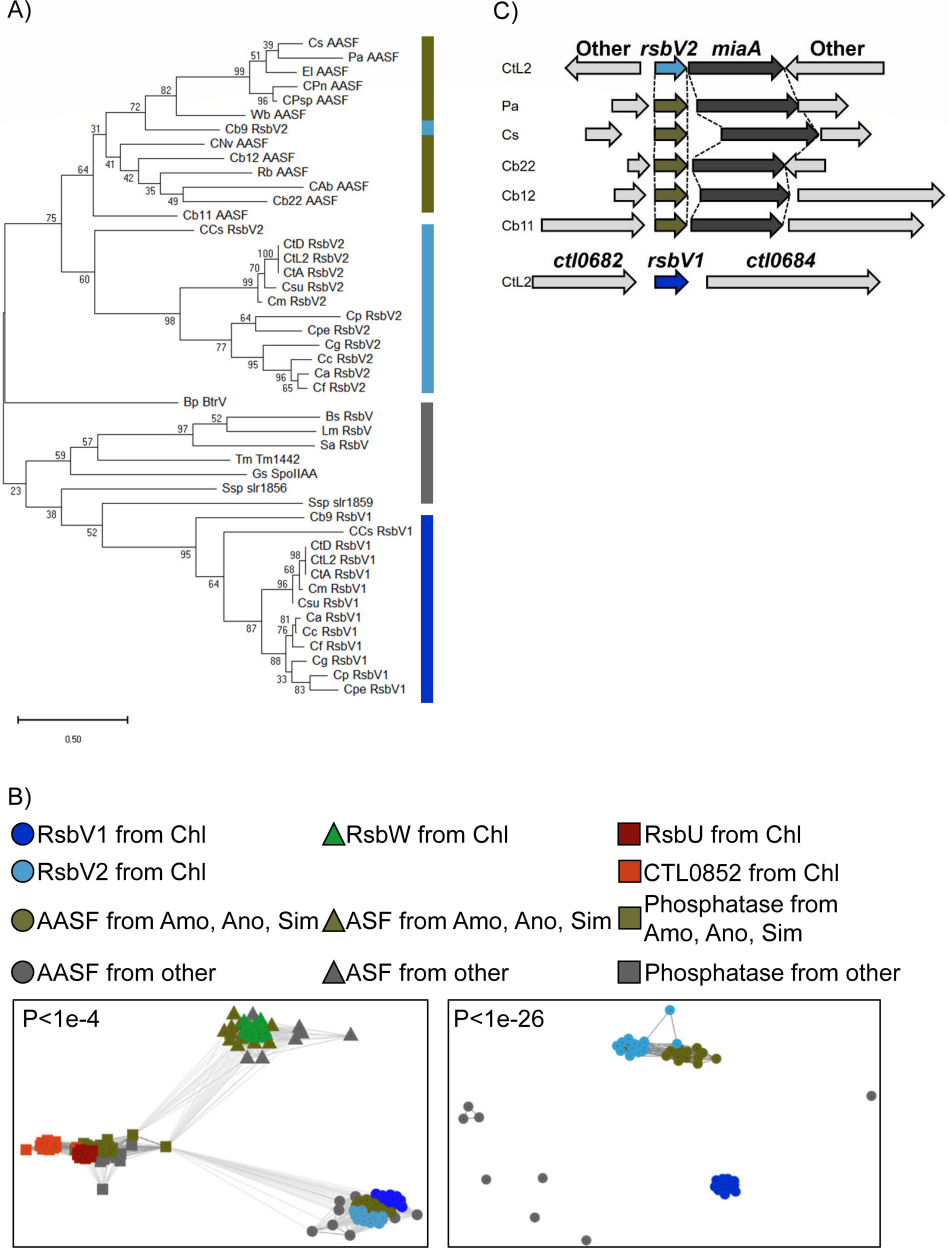
*In silico* studies reveal a potential AASF expansion. PSM homologs (RsbV, RsbW, and RsbU) were obtained from Chlamydiales (Chl), Amoebachlamydiales (Amo), Anoxychlamydiales (Ano), Simkaniales (Sim), and non-*Chlamydia* species (Other) for *in silico* analyses. NCBI IDs of each protein and species abbreviations are listed in Table S2. (**A**) A phylogenetic tree of AASFs was constructed in MEGA 11 using the maximum likelihood method with 1,000 bootstraps. Numbers represent the percentage of trees in which the associated taxa clustered together. (**B**) Protein sequence relationships were also analyzed in CLANS. The left panel describes PSM proteins with the connection defined as *P* < 1e^−4^, while the right panel shows AASF connections only using a *P* < 1e^−26^. (**C**) Gene synteny analysis of *rsbV2* (*rsbV*). Gene loci were obtained from the NCBI genome database (see Table S2 for NCBI IDs).

*C. trachomatis* and other members of the order Chlamydiales, in which the pathogenic *Chlamydia* species belongs (pathogens of animals, birds, and humans), possess two copies of RsbV, denoted RsbV1 and RsbV2. Possession of dual AASFs is atypical in bacteria, and is especially intriguing for a minimal genome organism such as *Chlamydia* (~1 to 1.3 Mbp) (3). Recently, a phylum-wide analysis revealed that the presence of dual AASFs is not universal across Chlamydiota, and most phylum members only have a single AASF (5). Given the divergence in genome conservation, we sought to identify which AASF represented the “ancestral” copy and why *Chlamydia* possess two copies. Phylogenetic (Fig. 2A) and CLuster ANalysis of Sequences (CLANS) analyses (Fig. 2B) along with gene synteny (Fig. 2C) suggest that the RsbV2 in pathogenic *Chlamydia* is more similar to the single AASF, RsbV, found in non-Chlamydiales members. This would make RsbV1 unique to pathogenic *Chlamydia* and suggests that RsbV1 may have served as an “expansion factor” involved in the leap to becoming an animal pathogen. In addition, the amino acid sequence identity of RsbV2/RsbV across species shows more variability than when comparing RsbV1 across species (Fig. S1; Table S1). We speculate that the RsbV2/V variability is linked to the longer evolutionary possession of this AASF and the diverse environments/hosts associated with these bacteria. For example, comparing *C. trachomatis* and *Chlamydia pneumoniae,* RsbV1 exhibits 71% similarity while RsbV2 has only 44% similarity. Relevant to our proposed dichotomy regarding the number of AASFs between pathogenic and “non-pathogenic” *Chlamydia*, there are some discrepancies in the literature and annotated genomes (5). While the following pathogenic *Chlamydia* have been annotated as possessing one AASF, our reassessment of the deposited genome sequences supports the presence of two AASFs for *Chlamydia abortus*, *Chlamydia pecorum*, *Chlamydia ibidis*, and *Candidatus* Clavichlamydia salmonicola (Table S2). Additionally, there are two “non-pathogenic” species annotated to have two copies of AASFs from the Amoebachlamydiae, *Parachlamydiaceae* bacterium HS-T3 and *Parachlamydia* sp. C2. The phylogenic tree (Fig. 2A) shows that both of these AASFs are more similar to RsbV2 than RsbV1.

So why do pathogenic *Chlamydia* maintain two AASFs? Our group and others have shown that both RsbV1 and RsbV2 can be phosphorylated by RsbW (38–40) on conserved serine residues, S56 and S55 [(39) (RsbV1), (40) (RsbV1/RsbV2)], respectively, in *C. trachomatis* and that both AASFs can be dephosphorylated by RsbU [(37, 39) (RsbV1/RsbV2); (38) (RsbV1)]. However, RsbV1 is a more favorable substrate of phosphorylation (38) and dephosphorylation (37–39) *in vitro* compared to RsbV2, and a mechanistic explanation for those differences is lacking. While the phosphorylation site motif in the AASFs is highly conserved (Fig. S1, Y[M/I/L]S**S**AG[I/V/M/L] across Chlamydiota, YMS**S**AGL for RsbV2 and YIS**S**AGI for RsbV1 in *C. trachomatis*), the models indicate differences in a number of amino acids at the RsbW-AASF interface that would alter the charge profile, potentially impacting RsbW-AASF binding and/or phosphorylation between RsbV1 and RsbV2 (Fig. S1 and S2). RsbV1 also has an additional six C-terminal amino acids (116 amino acids) versus RsbV2 (110 amino acids), and the proteins have predicted pI’s that vary by two logs [pI of 5 for RsbV1 (negatively charged in the chlamydial cytoplasm) and a pI of 7 for RsbV2 (no net charge), (47)]. We attempted to directly assess the importance of amino acids predicted to be involved in RsbW-AASF binding using amino acid point mutants, but the recombinant proteins were either insoluble or did not properly fold as measured by limited trypsin proteolysis (data not shown). Although the chlamydial cytoplasm has a pH of 7.28 (48), previous enzyme assays showing kinase and phosphatase kinetic differences were performed at pH 7.5 (37–39) or pH 7.6 (40). To see if the prior results showing differences for RsbV1 and RsbV2 were due to the more basic pH values, we assessed time course kinase and phosphatase assays at pH 7.28 and pH 7.5. Similar trends were observed as previously reported, with RsbV1 functioning as a preferred kinase (Fig. S3A) (38) and phosphatase substrate (Fig. S3B) (37–39) at both pH values. Overall, these data show that RsbV1 is unique to the pathogenic *Chlamydia* and confirmed that there are distinct biochemical differences between the AASFs when assayed *in vitro*, prompting us to investigate the physiological impact of each AASF *in vivo*.

### RsbV1/RsbV2 protein abundance has minimal impact on EB production

**Fig 3:**
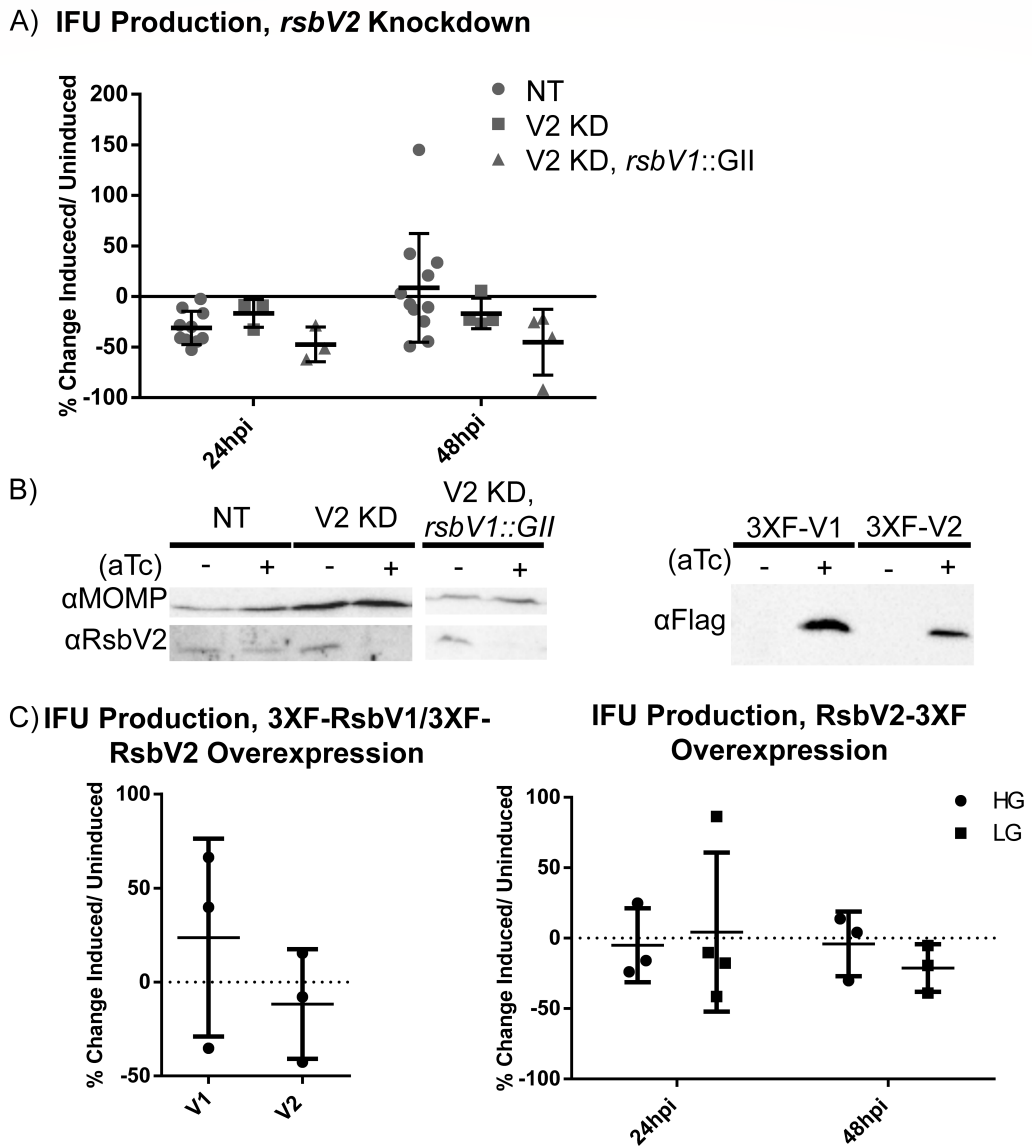
RsbV2 overexpression or *rsbV2* knockdown does not alter IFU production. Recombinant *C. trachomatis* L2 [wild-type chromosome or an *rsbV1*-inactivated strain (*rsbV1*::GII)] with CRISPRi dCas12 non-targeted (NT) or *rsbV2*-targeted (V2 KD) plasmids were used to infect HeLa cells. Knockdown of *rsbV2* was induced by 2 nM anhydrotetracycline (aTc) at 4 hpi, and samples were harvested at 24 or 48 hpi for infectious EB determination by IFU assay (**A**). Bars represent the mean, and the error bars report standard deviation. Statistical analysis was done by two-way analysis of variance; there is no significant difference. (**B**) Western blotting was performed to confirm RsbV2 knockdown (left panel) or RsbV2/RsbV1 overexpression (right panel). The Major Outer Membrane Protein (MOMP) was used as a western blot loading control. Primary antibodies used are indicated to the left of each blot. (**C**) N-ter 3XFLAG-tagged RsbV1 or RsbV2 was induced with 10 nM aTc in high glucose medium, and samples were harvested at 48 hpi for titering (left panel). C-ter 3XFLAG-tagged RsbV2 was induced with 10 nM aTc at 4 hpi in infected cells cultured in either high glucose or low glucose, and samples were harvested at 24 hpi or 48 hpi for titering by IFU assay (right panel). Bars represent the mean, and error bars report the standard deviation. Statistical analysis was done by *t*-test, and there is no significant difference. IFUs were normalized to uninduced samples for (**A**) and (**C**).

To assess the impact of each AASF on chlamydial growth, we employed knockdown and overexpression approaches. Previous work with an RsbV1-null strain (*rsbV1*::GII intron) showed that inclusion forming unit (IFU) production dropped by ~1 log compared to the parental strain (38, 39). Attempts to generate an *rsbV2*-null mutant have been unsuccessful, so we utilized CRISPR interference (CRISPRi)-mediated dCas12 knockdown of *rsbV2* as an alternative approach (49). We also overexpressed FLAG-tagged RsbV2 to assess the impact of increased levels of RsbV2 on chlamydial development. Knockdown of *rsbV2* in RsbV1-competent or RsbV1-null strains did not significantly reduce IFU production at 24 hpi compared to non-targeting and uninduced controls (Fig. 3A). At 48 hpi, a downward trend in IFU production was observed upon *rsbV2* knockdown for both the RsbV1-competent and RsbV1-null strains, but the differences were not statistically significant (Fig. 3A). RsbV2 western blots confirmed knockdown of *rsbV2* (Fig. 3B). Since significant changes were not observed in inclusion morphology (data not shown) or in IFU production, we did not measure changes in *miaA* levels (putative operon with *rsbV2*, Fig. 2C) or perform complementation experiments.

We next assessed the impact of overexpression of FLAG-tagged RsbV2 under high glucose or low glucose medium conditions, which resulted in no significant changes in IFU production (Fig. 3C). Consistent with prior results, overexpression of RsbV1 (as FLAG-RsbV1 in this study) resulted in an upward trend in IFU production. FLAG-tagged RsbV1 and RsbV2 expressions were confirmed by western blot (Fig. 3B; Fig. S4). Growth under different glucose conditions was tested as the PSM may function to set a growth cap and/or govern a developmental timeline in response to the levels of metabolites such as ATP (38–40), α-ketoglutarate (36), and phosphoenolpyruvate (PEP) (37). In characterized stress response PSMs, the proteins are induced whenever needed during stress exposure (9, 11, 31, 50); however, this does not seem to be the case in *C. trachomatis*. We previously reported that native RsbV1 and RsbV2 protein levels do not appear to shift relative to MOMP under different glucose levels and that RsbV2 levels do not change in an RsbV1-null mutant (39). Here, we show that there are minimal impacts on chlamydial growth when artificially modulating AASF protein levels, so we next sought to determine if phosphorylation status is more relevant than protein abundance.

### Expression of a non-phosphorylatable RsbV1 S56A mutant partially complements an RsbV1-null mutant, but has no impact in a wild-type background

Soules et al. found that an RsbU-null strain (PSM phosphatase deficient) was severely attenuated for growth and IFU production compared to wild-type and *rsbU*-complemented strains (36). In addition, Fisher et al. detected phosphorylated RsbV1 and RsbV2 in EBs, but not in RBs, for *Chlamydia caviae* (51), showing that phosphorylation might vary across development. These data and our data support that AASF phosphorylation status may be more physiologically relevant than AASF abundance for PSM regulation. Previous data from surface-plasmon resonance experiments (38) and yeast two-hybrid (40) or bacterial adenylate cyclase two-hybrid experiments [data not shown, (52)] demonstrated that RsbW forms a more stable complex with the RsbV1 S56A and RsbV2 S55A mutants than with the wild-type proteins. “Trapping” of RsbW by an AASF should increase the pool of free σ^66^. Theoretically, free σ^66^ leads to increased competition with late-gene sigma factors (σ^54/28^) for binding with the RNAP core and/or alterations in transcription of σ^66^-dependent genes via promoter titration. Competition and promoter titration would be predicted to interfere with growth and/or secondary differentiation. To assess the importance of reversible AASF-RsbW interactions, we expressed non-phosphorylatable RsbV1 (S56A) and RsbV2 (S55A) analogs.

To confirm that RsbW is active in *C. trachomatis* during development, we overexpressed a FLAG-tagged RsbV1 and used Phos-tag acrylamide western blot to assess RsbV1 phosphorylation status. Phosphorylated RsbV1 was detected at all time points tested (Fig. S4) confirming that RsbW is active during infection and that the FLAG-tag does not prevent RsbW-RsbV1 interactions and phosphorylation *in vivo*. We were surprised that only phosphorylated FLAG-tagged RsbV1 was detected and suspect that either technical limitations associated with Phos-tag western blots (uneven transfer of proteins) or tag interference with normal RsbU phosphatase kinetics obscured the expected variable phosphorylation of RsbV1 (RsbV1 early and P-RsbV1 late). Our native-RsbV1 antibodies were not sufficient to detect either species. Alternative approaches such as phospho-specific antibodies may be needed to determine whether phosphorylation is variable under the conditions tested.

**Fig 4:**
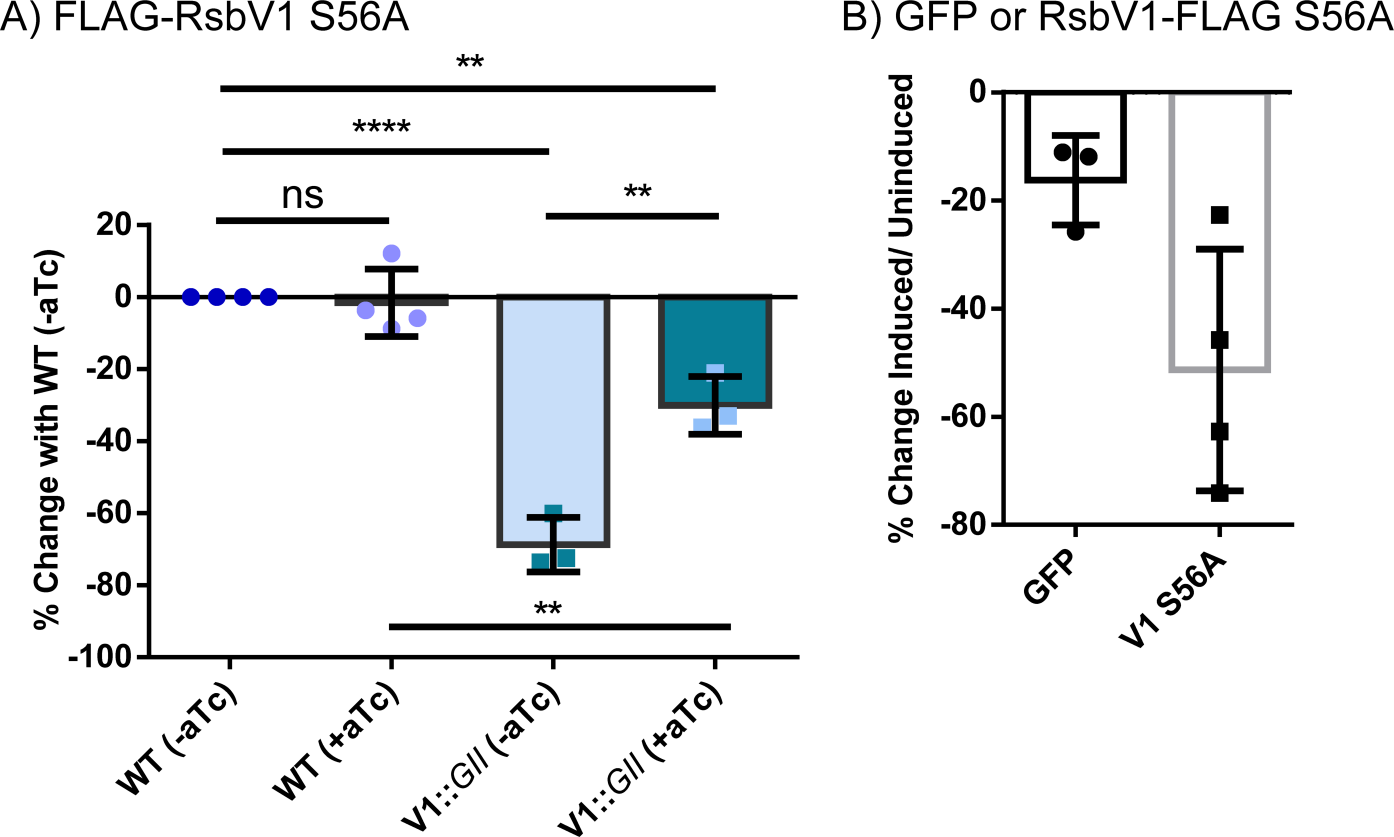
Induction of a non-phosphorylatable RsbV1 S56A mutant does not alter IFU production. RsbV1 S56A was induced with aTc at 4 hpi in recombinant *C. trachomatis* L2 [wild-type chromosome or an *rsbV1*-inactivated strain (*rsbV1*::GII)]. Titers were assessed for infectious progeny numbers at 48 hpi using the IFU assay. (**A**) N-ter 3XFLAG-tagged RsbV1 S56A induced with 10 nM aTc, (**B**) C-ter 3XFLAG-tagged GFP (control) or RsbV1 S56A induced with 112 nM aTc. Statistical analysis shows no significant difference. Statistical analysis was done with the *t*-test (ns = *P* > 0.05, ** = *P* ≤ 0.01, **** = *P* ≤ 0.0001).

In conflict with our RsbW-trapping hypothesis, expression of an N-terminal FLAG-RsbV1 S56A mutant in a wild-type background strain resulted in no significant differences in IFU production at 24 (data not shown) or 48 hpi (Fig. 4A). Unexpectedly, FLAG-RsbV1 S56A expression in the RsbV1-null strain resulted in a significant increase in IFU production of ~50% at both time points. To assess if the FLAG-tag location impacted the results for RsbV1 S56A, we also tested a C-terminal FLAG-tagged RsbV1 S56A. For reasons that we cannot explain, expression of the C-terminal-tagged construct was not detectable via anti-FLAG western blot even with increased anhydrotetracycline (aTc)-inducer levels (112 nM vs 10 nM used in other experiments). RsbV1-FLAG S56A expression could be detected via immunofluorescence, although the amount of RsbV1-FLAG S56A produced was well below the levels of RbsV2-FLAG S55A expressed under equivalent induction conditions (Fig. S5). Expression of the RsbV1-FLAG S56A did result in a 50% reduction in IFUs compared to the uninduced strain. However, the reduction was not significantly different when compared to a GFP-FLAG expressing strain used as a control for the high concentration of aTc used for induction, which can inhibit chlamydial growth (53) (Fig. 4B).

### Expression of a non-phosphorylatable RsbV2 S55A mutant significantly reduces IFU production, but not bacterial numbers

**Fig 5:**
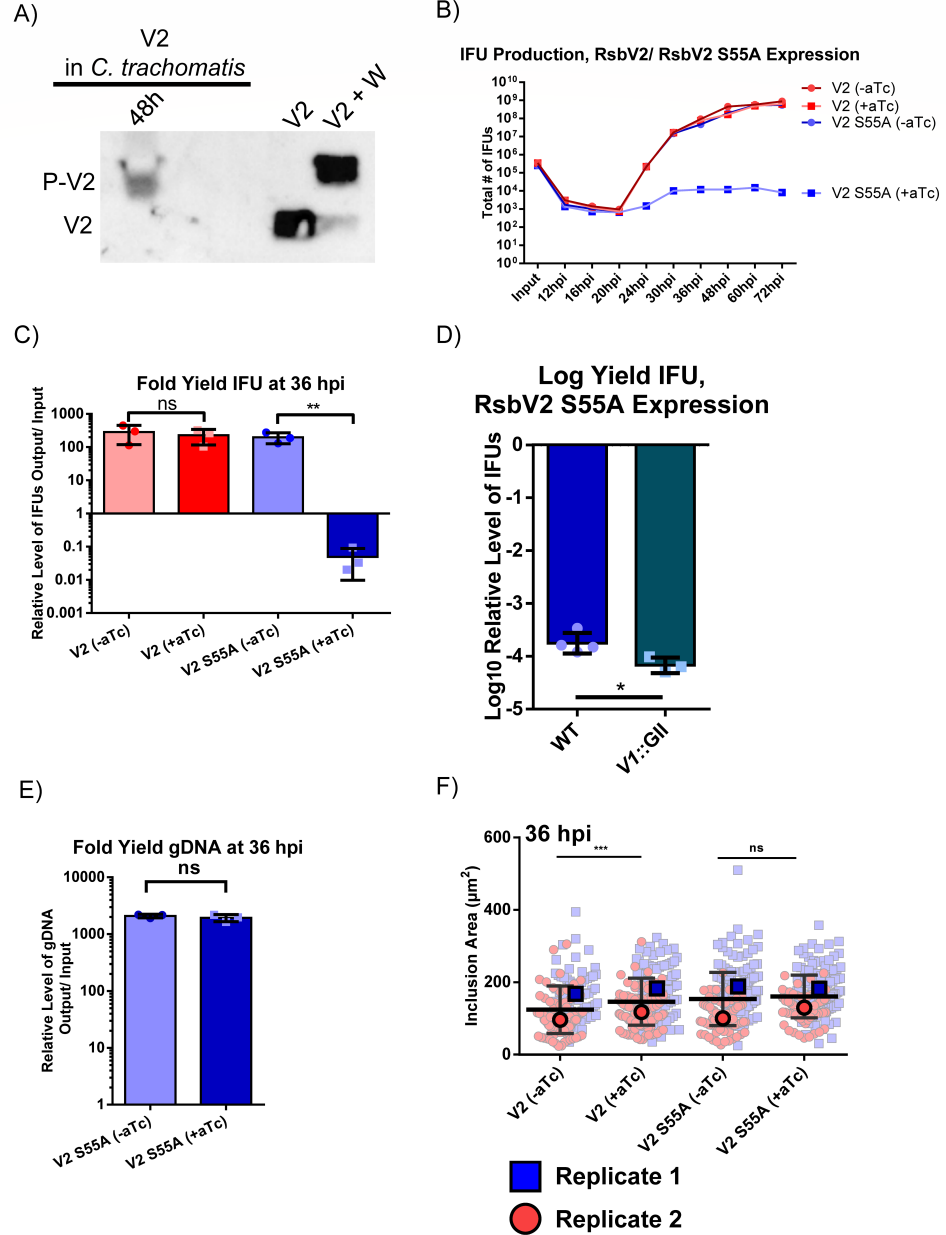
Impact of phosphorylation/non-phosphorylation status of RsbV2 on chlamydial growth and cell development. (**A**) *C. trachomatis* L2-infected HeLa cells were harvested at 48 hpi. RsbV2 was immunoprecipitated with an anti-RsbV2 antibody. Phosphorylation status of endogenous RsbV2 was assessed by running samples on 16% Phos-tag SDS-PAGE gels followed by western blot with an anti-RsbV2 antibody. *E. coli* coexpression lysates [RsbV2 plus empty vector (V2) or RsbV2 plus His-tagged RsbW (V2 + W)] were used as negative and positive controls for RsbV2 phosphorylation, respectively. Expected protein positions are listed to the left of the blot. (**B**) Recombinant *C. trachomatis* L2 with an aTc-inducible C-ter 3XFLAG-tagged wild type or RsbV2 S55A were used to infect HeLa cells. RsbV2 was induced by adding 10 nM aTc at 4 hpi. Samples were harvested at different time points, and infectious EBs titers were determined using an IFU assay. Fold yield for the 36 hpi time point is reported in (**C**). Note that the IFU fold yield for the induced RsbV2 S55A mutant strain was significantly different at 36–72 hpi, while the induced wild-type RsbV2 strain was not. (**D**) C-ter 3XFLAG-tagged RsbV2 S55A was induced with 4 nM aTc in a wild-type chromosome background or an *rsbV1*-inactivated strain (*rsbV1*::GII) at 4 hpi, and harvested at 48 hpi. (**E**) The number of bacteria (RBs + EBs) at 36 hpi were quantified by quantitative PCR (qPCR) and represented with fold yield of gDNA. Plot in (**B**) is representative of three experiments, (**C**), (**D**), and (**E**) report the averages for all three trials. (**F**) Immunofluorescence microscopy was performed with anti-MOMP antibody and an anti-mouse secondary conjugated with Alexa Fluor 488 to demarcate inclusions. Images were acquired at 1,000× magnification and the size of inclusions was measured using ImageJ. Pale symbols report individual inclusion size, dark symbol represents the average of each replicate. Statistical analyses were done with the *t*-test (ns = *P* > 0.05, * = *P* ≤ 0.05, ** = *P* ≤ 0.01, *** = *P* ≤ 0.001).

Using a similar approach as with RsbV1, we first attempted to detect phosphorylated RsbV2. Attempts to detect endogenous RsbV2 using Phos-tag acrylamide gels and anti-RsbV2 western blot were only successful with samples harvested at 48 hpi (EB-enriched) and detection required RsbV2 immunoprecipitation from bacteria-enriched samples (Fig. 5A). To explore earlier events, we used an RsbV2-FLAG construct. Only phosphorylated RsbV2-FLAG was detected (Fig. S4B) as seen for FLAG-RsbV1. *In vitro* kinase and phosphatase assays with N-terminal and C-terminal FLAG-tagged RsbV2 showed that phosphatase activity was impaired compared to activity toward His-P-RsbV2 (Fig. S4C and D). Nevertheless, we documented that RsbW can phosphorylate FLAG-RsbV2 *in vivo*.

In contrast to the results from the RsbV1 S56A overexpression experiments, expression of an RsbV2-FLAG S55A mutant resulted in a significant drop in IFU production with an approximate 3 log reduction observed at 36 hpi and beyond compared to the uninduced sample or a strain overexpressing wild-type RsbV2-FLAG (Fig. 5B and C). Allowing infections to proceed for 72 hpi did not lead to recovery of IFU numbers. Since expression of the RsbV1 S56A in the RsbV1-null strain had a modest rescuing effect on IFU production comparing wild-type and RsbV1-null IFU production levels, we expressed RsbV2-FLAG S55A in the RsbV1-null background. In contrast to the RsbV1 S56A results (rescue), IFU production was decreased ~3 log when expressing the RsbV2-FLAG S55A in the RsbV1-null background (Fig. 5D), and the reduction in IFUs was greater than when expressing RsbV2-FLAG S55A in the wild-type background (RsbV1/RsbV2 competent strain).

The severe IFU reduction resulting from RsbV2 S55A overexpression led us to further define the phenotype. In contrast to the significant reduction in IFUs, using quantification of genomic DNA as a surrogate for bacterial numbers, we observed no significant difference between RsbV2 S55A-induced and uninduced samples at 36 hpi [Fig. 5E, similar results at 24 and 48 h (data not shown)]. In addition to equivalent genomic DNA levels, inclusion sizes measured using immunofluorescence microscopy were not grossly altered when expressing RsbV2-FLAG S55A [no significant differences at 24 hpi (Fig. S6B) or 36 hpi (Fig. 5F)], with a modest size increase found at 48 hpi (Fig. S6B). Because of potential FLAG-tag impacts, we also performed experiments with an N-terminal His-tagged RsbV2 S55A mutant. The results phenocopied the IFU results for the RsbV2-FLAG S55A mutant (Fig. S7). The impact of RsbV2 S55A expression on growth under low glucose conditions could not be measured owing to the already reduced IFU production of the uninduced strain during low glucose conditions (data not shown).

### Expression of RsbV2 S55A blocks chlamydial developmental

**Fig 6:**
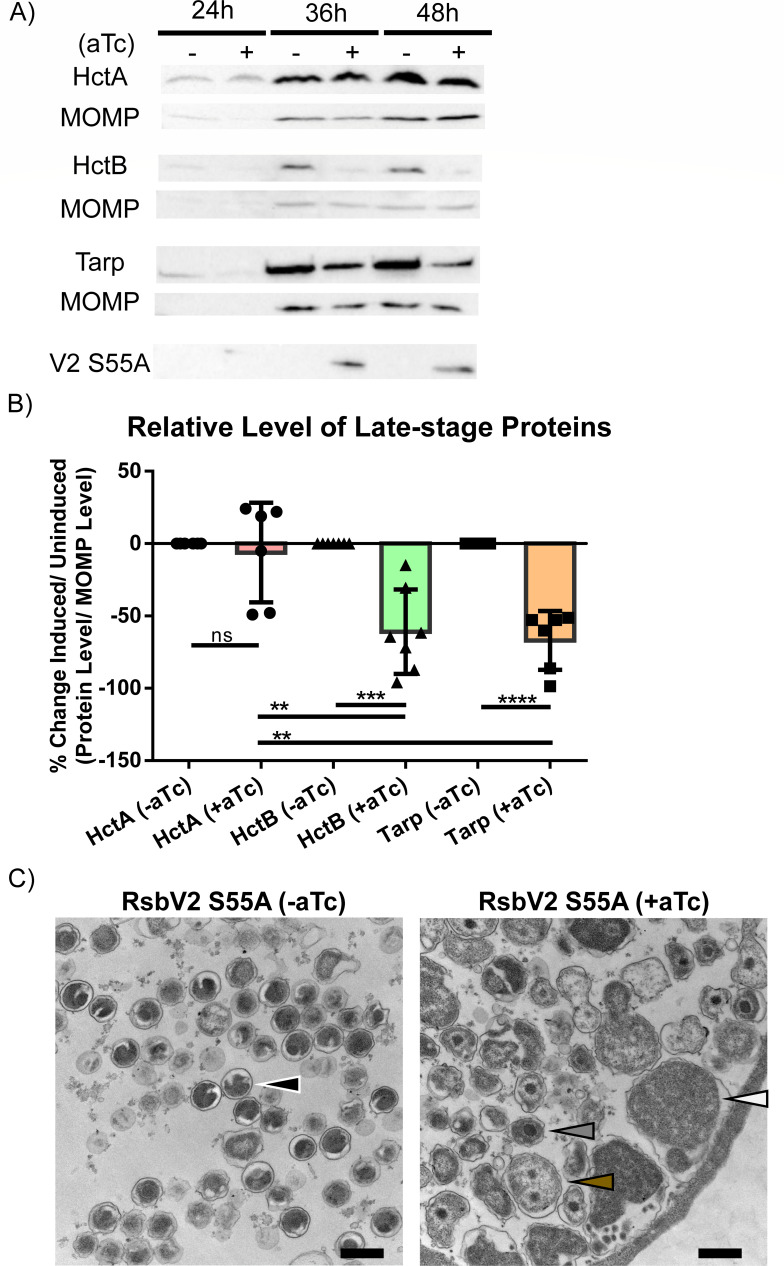
Induction of the RsbV2 S55A mutant reduces production of late-stage proteins and reduces EB formation. Recombinant *C. trachomatis* L2 producing C-ter 3XFLAG-tagged RsbV2 S55A was used to infect HeLa cells. RsbV2 was induced by adding 10 nM aTc, and lysates were harvested at 24 hpi, 36 hpi, 48 hpi for western blot or samples were fixed at 48 hpi for transmission electron microscopy (TEM). (**A**) Protein production was determined using quantitative western blot of induced or uninduced RsbV2 S55A samples. MOMP was used as a loading control. Proteins detected are listed to the left of each blot. (**B**) Protein levels were quantified in reference to MOMP for HctA, HctB, and Tarp. Statistical analysis was done with the *t*-test by using HctA as a reference. (ns = *P* > 0.05, ** = *P* ≤ 0.01, *** = *P* ≤ 0.001, **** = *P* ≤ 0.0001). (C) Uninduced (left image) or induced (right image) sample images were taken by TEM with magnification at 26,000×. Arrows denote a representative normal EB (black), IB (gray), abnormal IB (brown), and an RB (white). Scale bars are 500 nm. Additional TEM images are shown in Fig. S8.

The reduction in IFUs without gross alterations in inclusion morphology or reduced levels of genomic DNA indicated that chlamydial development was being perturbed with either lack of EB production and/or the production of non-infectious EBs. We initially used western blot to determine if production of late-stage proteins had been altered (Fig. 6A). We assessed three late proteins regulated by the three different sigma factors: the histone-like proteins HctA [regulated by σ^66^ (54)] and HctB [regulated by σ^28^ (41, 44)] and the type 3 secretion effector Tarp [regulated indirectly by σ^54^ (43, 44)]. Protein levels were compared to MOMP as a marker for total bacteria (Fig. 6B). For HctA, there was no difference when comparing uninduced and induced samples. However, levels of both HctB and Tarp were significantly reduced under induced conditions. These results are consistent with reduced amounts of RNAP-σ^54/28^ holoenzyme complexes or reduced levels of σ^54^/^28^ altogether, and suggest that EB production is altered or blocked in the presence of RsbV2 S55A. We next used transmission electron microscopy to visualize the bacteria within the inclusion. Samples were imaged at 48 hpi, and inclusions from induced samples showed relatively few EBs, numerous intermediate bodies (IBs) and atypical IBs, and numerous RBs, whereas uninduced samples contained mostly EBs (Fig. 6C; Fig. S8). In whole, our data support that expression of RsbV2 S55A leads to disruption of secondary differentiation with bacteria appearing to either remain as RBs or stall at the IB stage, which is representative of early secondary differentiation events. Extrapolating the protein production data for HctB and Tarp to other late proteins, we predict that secondary differentiation cannot be completed owing to reductions in σ^54/28^-regulated gene products.

## DISCUSSION

During chlamydial evolution, genome reduction has been balanced with gene acquisition allowing for select chlamydial species to gain the ability to infect vertebrates (5). One such expansion appears to have occurred within the chlamydial Rsb-like partner switching mechanism whereby the pathogenic *Chlamydia* and other Chlamydiales members possess two AASF homologs, RsbV1 and RsbV2, while the majority of the phylum Chlamydiota have a single AASF. CLANS, phylogeny, gene synteny, and sequence comparisons support that RsbV2 is the “ancestral” RsbV, whereas RsbV1 is the acquired AASF. Note that we cannot rule out that both copies existed in a last universal common ancestor with RsbV1 being deleted in non-Chlamydiales members. It is also unclear as to where *rsbV1* would have originated given the somewhat “sheltered” life for obligate intracellular bacteria. We hypothesize that dual AASFs played a role in the leap to infecting vertebrates, and both are found in the fish pathogen *Candidatus* Clavichlamydia salmonicola (along with the late gene regulator σ^28^ and various type 3 secretion effector proteins), a non-Chlamydiaceae member that has been proposed to be a transitional strain between environmental and pathogenic *Chlamydia* (4).

We remain left with the question as to what benefit a second AASF provides and how this altered PSM network facilitates bacterial infection in vertebrates. RsbV1 and RsbV2 have different biochemical properties with disparate pI’s (5 and 7, respectively) and altered amino acids at the predicted AASF/ASF interaction site. Despite using similar approaches, the *in vivo* cell culture study reveals conflicting results between RsbV1 and RsbV2. Inactivation of RsbV1 or knockdown of RsbV2 shows either a ~1 log reduction in EB production or no significant reduction in EB production, respectively (38, 39). Overexpression of either RsbV1 or RsbV2 also fails to elicit significant phenotypic changes. To perturb phosphorylation-mediated regulation of the AASFs, we overexpressed non-phosphorylatable forms of RsbV1 (S56A) and RsbV2 (S55A). While we did not observe significant phenotypes when overexpressing RsbV1 S56A in a wild-type background, overexpression of RsbV2 S55A resulted in ~3 log reduction in IFUs. Interestingly, the induction of these mutants in the RsbV1-inactivated strain provided varying results: partial rescue with RsbV1 S56A induction or exacerbated IFU reductions upon RsbV2 S55A induction. The fact that a similar approach yields different results between RsbV1 and RsbV2 highlights their non-redundancy and unique physiological roles in *C. trachomatis*. There is evidence for differential impacts of AASF deletion on bacterial phenotypes. Like the pathogenic *Chlamydia* that possess two AASFs (RsbV1 and RsbV2), the cyanobacterium *Synechocystis* sp. PCC 6803 has two AASFs with Slr1856 a better *in vitro* RsbW substrate than Slr1859, and Slr1859 is not dephosphorylated *in vitro* by the RsbU homolog IcfG (55). Deletion of the “inferior” AASF, Slr1859, results in attenuated growth in inorganic carbon-limited medium supplemented with glucose, whereas a Slr1856 deletion does not impact growth (56).

**Fig 7:**
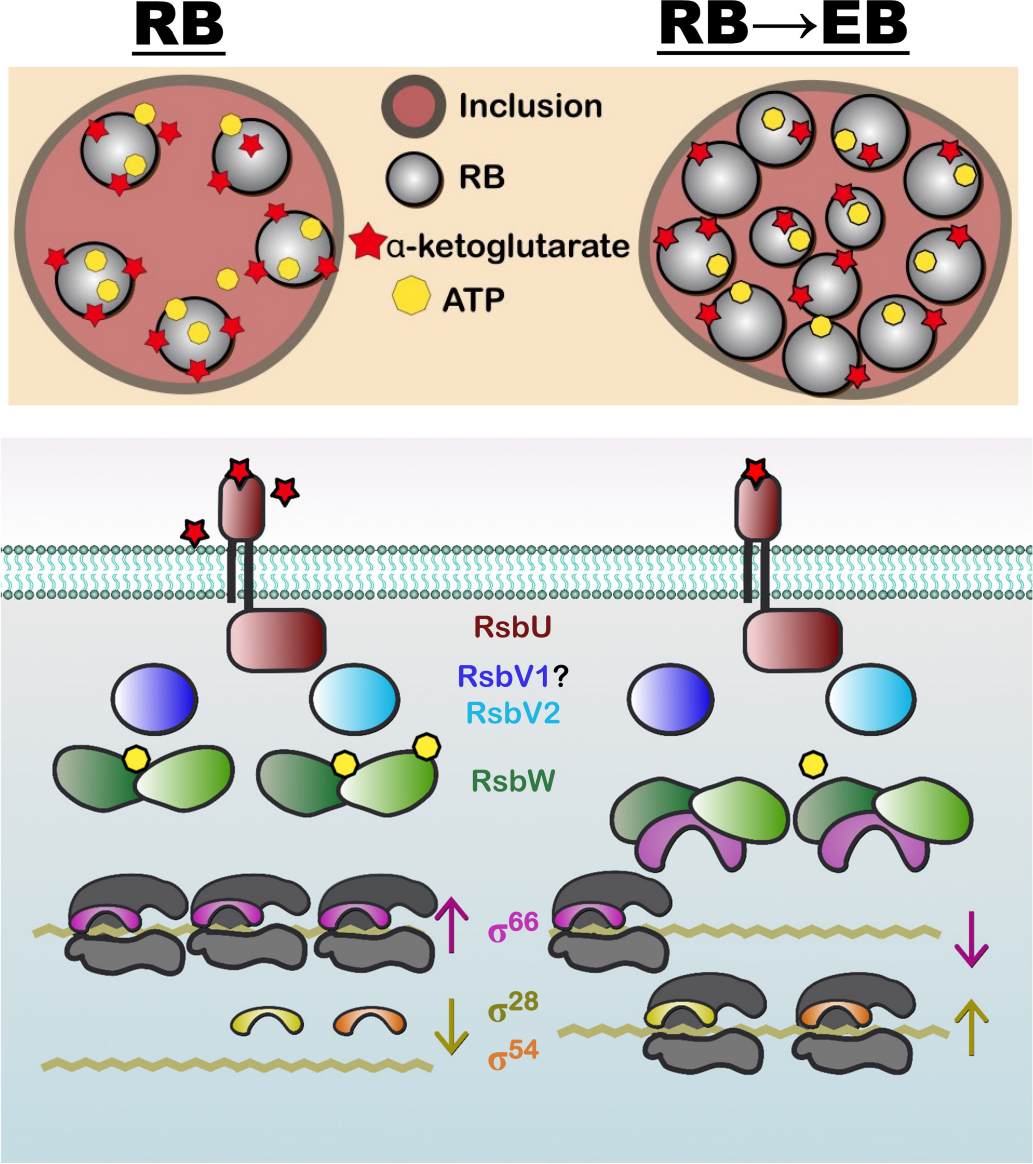
A working model of PSM-mediated developmental regulation. RsbW interacts with σ^66^ and represses transcription, or it phosphorylates RsbV1 and RsbV2 leading to derepression in an ATP-dependent manner. RsbU dephosphorylates both RsbV1 and RsbV2 in response to cell-derived, periplasmic α-ketoglutarate (prediction). During growth and replication, bacteria compete for nutrients including cell-derived ATP, glucose-6-P (can be used for substrate-level phosphorylation), and α-ketoglutarate. As bacterial numbers increase, the reduction of ATP and α-ketoglutarate levels decrease the signal to RsbU and RsbW leading to RsbW sequestering a pool of σ^66^, allowing alternative sigma factors (σ^28^ and σ^54^) to access the RNAP core supporting RB to EB conversion.

We were initially surprised to observe a ~3 log reduction in IFUs for the RsbV2 S55A overexpression strain as inclusion size was not obviously impacted. Quantification of genomic DNA under induced and uninduced conditions suggested that total number of bacteria were also similar in the presence or absence of exogenous RsbV2 S55A. Our model predicts that RsbV2 S55A should bind and sequester RsbW, freeing σ^66^ and leading to dysregulation of secondary differentiation. Potential mechanisms underlying dysregulation include a reduction in late gene expression through alterations in the levels of σ^54^ and/or σ^28^ or reduction in σ^54^/σ^28^-RNAP complexes due to competition with σ^66^ (Fig. 7). Knockdown of σ^28^ and/or σ^54^ has been shown to be detrimental for IFU production, which phenocopies our results when overexpressing RsbV2 S55A (44).

Consistent with a block in secondary differentiation, western blot quantification of σ^54^- and σ^28^-regulated proteins [Tarp (indirect) and HctB, respectively] showed reduced levels while the σ^66^-regulated late protein HctA levels were normal. Note that previous transcription experiments looking at *hctB* expression when altering PSM component levels did not show alterations (36, 38). Those experiments looked at earlier time points (12, 18, 24, and 30 hpi) than our study (48 hpi). TEM images of infected cells corroborated a developmental block in RsbV2 S55A expressing bacteria, which were predominantly in the RB or IB form compared to matched, uninduced samples that were enriched with EBs. The block in differentiation from IB to EB suggests that secondary differentiation has commenced in RsbV2 S55A conditions, likely through σ^66^-regulated late gene products such as HctA, but the production of σ^54^- and σ^28^-regulated late genes has not occurred to enable completion of secondary differentiation. Our results confirm the hierarchy of differentiation with σ^66^-regulated genes initiating secondary differentiation followed by the σ^54^ and σ^28^ regulons (57). Knockdown of RsbV2 had no effect on chlamydial growth, but knockdown is not equivalent to a gene knockout. Attempts to knockout *rsbV2* have been unsuccessful and could be attributed to technical problems, gene essentiality, or operon disruption as *rsbV2* is 5´ to *miaA* [tRNA dimethylallyltransferase, not essential in other bacteria (58)].

The absence of a phenotype when overexpressing RsbV1 S56A remains perplexing. RsbV1 S56A is a better binding partner for RsbW *in vitro*, and overexpressing the lower affinity RsbV2 S55A resulted in a very strong, deleterious phenotype (3 log reduction in IFUs). A previous study using an *rsbV1*-null strain shows a potential reduction in secondary differentiation (a 1 log IFU reduction). Here, we find partial complementation of the *rsbV1*-null strain upon RsbV1 S56A induction. To explain these observations, we propose that RsbV1 may not be able to disrupt σ^66^-RsbW binding and that RsbV1 may have another target. There is evidence in other bacteria that the non-phosphorylated/phosphorylated AASF is involved in cell signaling and regulation of other factors beyond the ASF. In *Sinorhizobium meliloti*, the phosphorylated AASF promotes the synthesis of mixed-linkage β-glucans through activation of a diguanylate cyclase by an unknown mechanism (59), and the non-traditional AASF from *Pseudomonas aeruginosa* interacts with FlgM in its non-phosphorylated form and c-di-GMP synthase in the phosphorylated form (18, 60, 61). There are additional examples of an AASF potentially moonlighting outside of the PSM including regulation of the type 3 secretion system in *Bordetella* spp. (62), hormogonia motility development in *Nostoc punctiforme* (19), biofilm formation in *Vibrio fischeri* (63), and production of a gene transfer agent (phage-like particle) and stationary phase viability in *Rhodobacter capsulatus* (64). Similar to these other PSM systems, we hypothesize that RsbV1 has a moonlighting role and that its primary function may be to regulate an unknown target upon phosphorylation/dephosphorylation by RsbW and RsbU. Such a scenario would leave RsbV2 as the canonical AASF within the chlamydial PSM.

In summary, this study compared and contrasted two AASFs from the Rsb-like PSM in *C. trachomatis* using *in silico*, *in vitro*, and *in vivo* approaches. Our findings suggest that RsbV2 is the canonical AASF shared across the phylum Chlamydiota. It plays a part in regulating the availability of σ^66^ in an RNAP competition model with two late-gene alternative sigma factors. RsbV1 appears to have been acquired by pathogenic *Chlamydia* where it may have a moonlighting function important for infecting vertebrates. Together, our study supports a PSM-based mechanism for the regulation of cell differentiation in *C. trachomatis* through two AASFs with distinct physiological roles.

## MATERIALS AND METHODS

### Bioinformatic analyses

Protein sequences for PSM component homologs were obtained from the NCBI database (https://www.ncbi.nlm.nih.gov/, IDs listed in Table S2). AlphaFold-predicted protein structures were downloaded from UniProt (https://www.uniprot.org/). Crystal protein structures of homologous proteins from *Geobacillus stearothermophilus* were obtained from the PDB database [https://www.rcsb.org/, SpoIIAB-σB (1L0O) (29)] and SpoIIAB-SpoIIAA [1TID (32)]. Structures were visualized using UCSF chimera (65), and the AlphaFold-derived chlamydial protein structures were superimposed with the crystal structure template followed by removal of the template. Amino acid sequences for AASF homologs from multiple species were aligned using Multiple Sequence Comparison by Log-Expectation (https://www.ebi.ac.uk/Tools/msa/muscle/). The alignment was visualized in BioEdit version 7.2.5 using a 40% cutoff for similarity/identity (66). The phylogenetic tree was constructed using Molecular Evolutionary Genetics Analysis (MEGA 11) with maximum likelihood with 1,000 bootstraps (67) (https://www.megasoftware.net/). Sequence similarities were mapped using CLANS [(68), https://toolkit.tuebingen.mpg.de/tools/clans] using the BLOSUM62 scoring matrix and E-values of 1e^−4^. For the PSM protein map, proteins were connected using *P*< -1e^−4^, and *P*< -1e^−26^ was used for the for AASF protein map.

### Cell, chlamydial, and *E. coli* culture conditions

HeLa cells were routinely passaged in Dulbecco’s Modified Eagles Medium (DMEM) with 10% fetal bovine serum (FBS) at 37°C with 5% CO_2_. Cells were tested for mycoplasma contamination by PCR. A plasmid-free *C. trachomatis* L2 strain was used as the parent for recombinant strain construction (strains and vectors are listed in Table S3). Recombinant chlamydial strains were passaged in the presence of 500 µg/mL of spectinomycin for p2TK2-derived vectors (69) or 0.5 µg/mL of ampicillin for pBOMB-derived vectors (70), and titers were measured using the IFU assay (71) using intrinsic fluorescence from the vector-derived GFP (pBOMB-series) or RFP (p2TK2-series).

HeLa cells were infected with *C. trachomatis* at an multiplicity of infection (MOI) of 0.5 in infection medium (DMEM, 10% FBS, 1× non-essential amino acids, 400 ng/mL cycloheximide, and the appropriate antibiotic) for IFU and qPCR assays. An MOI of 0.25 was used for immunofluorescence assays with coverslips in 24-well plates. Infection was initiated through centrifugation at room temperature at 500 × *g* for 30 min for 24-well plates or 60 min for 96-well plates. When needed, *rsbV1*/*rsbV2* gene expression was induced by adding 10 nM aTc at 4 hpi except for experiments with C-terminal 3XFLAG-tagged RsbV1 S56A which used 112 nM aTc. For CRISPRi experiments, 2 nM of aTc was added at 4 hpi. To measure infectious progeny production from recombinant strains, 24-well plates were used. Well contents were harvested at 24 or 48 hpi by collection of supernatants followed by cell lysis via the addition of sterile water. Supernatant and lysis samples were pooled and bacteria were pelleted by centrifugation at 4°C at 13,000 × *g* for 5 min. Pellets were then suspended in 200 µL sucrose phosphate glutamate buffer (SPG) for RsbV2 (wild type and S55A) induction and 500 µL SPG for other strains, and stored at −80°C. To collect samples at multiple time points, 96-well plates were used as described for the 24-well plates. The lysed pellets were then suspended in 75 µL SPG for titering or 200 µL phosphate buffered saline (PBS) for qPCR, and stored at −80°C. For western blot samples, cells were infected at an MOI of 0.5, and samples were harvested at 24, 36, and 48 hpi from 24-well plates using 150 µL of Laemmli buffer with β-mercaptoethanol to directly lyse the cells followed by heating at 95°C for 5 min.

*E. coli* strains DH5a, XL-1 Blue, or NEB10 were used for cloning, while *E. coli* BL21(DE3) or NEB express was used for protein production. Bacteria were grown in lysogeny broth (LB) or on LB agar plates at 37°C supplemented with antibiotics as needed: 100 µg/mL ampicillin, 50 µg/mL spectinomycin, and/or 34 µg/mL chloramphenicol.

### Quantification of *C. trachomatis* gDNA

Infected HeLa cells were processed as described in reference (39). Genomic DNA was isolated, and 50 ng of purified DNA was mixed with 0.3 µM of *omcB* forward and reverse primers with Power SYBR Green PCR Master Mix (Applied Biosystems). The qPCR reactions were run on a QuantStudio 3 (Applied Biosystems). The data were normalized using a standard curve from gDNA extracted from purified EBs.

### Vector construction

Genes were amplified by PCR using Phusion MASTER Mix (Thermo Scientific) (primers are listed in Table S4) or obtained as synthetic gBlocks (Integrated DNA Technologies, Table S5) and inserted into vectors using standard restriction cloning procedures, NEBuilder HiFi DNA Assembly (New England BioLabs), or ligation-independent cloning (pLATE31). The pLATE31 (Thermo Scientific) and pACYC-1 duet (Novagen) vectors were obtained from commercial sources. The *E. coli-C. trachomatis* shuttle vector p2TK2_Spec_-SW2 mCh(Gro_L2_) Tet-IncV-3XFLAG (kindly gifted from Isabelle Derré) was used as a template for chlamydial expression vector construction (69). For gene knockdown, the pBOMBL12CRia::L2 vector was used (49). For site-directed mutagenesis, 5´-phosphorylated primers were used to amplify DNA followed by treatment with DpnI and self-ligation with T4 DNA ligase. All vectors were purified using the GeneJet Plasmid Kit (Thermo Scientific) and sequence verified by Sanger sequencing (PSOMAGEN). Vectors transformed into *C. trachomatis* were reisolated by transforming *E. coli* NEB10 with DNA isolated from recombinant strains (via the Whole Blood Genomic DNA Purification Kit, Thermo Scientific) followed by selection on LB agar with the appropriate drug. Plasmids were then isolated and checked for size on agarose gels, and the insert sequence reconfirmed by Sanger sequencing.

### Immunofluorescence microscopy

Cells were fixed at 24, 36, or 48 h (for 3XFLAG RsbV2/3XFLAG RsbV2 S55A induced with 10 nM aTc) or 48 h only (for 3XFLAG RsbV1 S56A/3XFLAG RsbV2 S56A induced with 112 nM aTc) in 3% paraformaldehyde and 3% sucrose in PBS and then permeabilized with 0.2% Triton X-100 in PBS. *Chlamydia* were detected using a mouse anti-MOMP primary antibody (1:250, Abcam ab41193) followed by staining with a donkey anti-mouse IgG-Alexa Fluor 488 secondary antibody (1:2,000, Invitrogen A11001). FLAG-tagged proteins were detected with an anti-FLAG antibody (1:500, Sigma F1804). Samples were also stained with 4′,6-diamidino-2-phenylindole (DAPI) (300 nM) to detect DNA. Images were taken with a Leica DMi8 at 1,000× with oil immersion and processed with ImageJ (NIH). Inclusion measurements were taken from at least two independent experiments, and a minimum of 90 inclusions were counted in total.

### Protein detection via western blot

Laemmli-treated, *C. trachomatis*-infected cell samples were resolved on 15% SDS-PAGE gels for RsbV1, RsbV2, and HctA, 12% SDS-PAGE gels for HctB, or 6% SDS-PAGE gels for Tarp. Detection of phosphorylated RsbV2 is detailed in the Supplemental Material. Protein was then transferred to nitrocellulose membranes followed by blocking with 5% milk-Tris buffered saline (MTBS) for 1 h at room temperature. Primary antibodies diluted in MTBS were used to probe blots overnight at 4°C: polyclonal rabbit anti-RsbV2 antibody (1:200) (39), polyclonal rabbit anti-RsbV1 antibody (1:200) (39), monoclonal mouse anti-FLAG antibody (Sigma F1804, 1:1,000), mouse anti-MOMP antibody (Abcam ab41193, 1:1,000 [1:100,000 for HctB quantification experiments]), rabbit anti-HctA antibody (kindly gifted by Ted Hackstadt, 1:1,000), rabbit anti-HctB antibody (kindly gifted by Ted Hackstadt, 1:1,000), or rabbit anti-Tarp antibody (kindly gifted by Ted Hackstadt, 1:100,000) (72). Blots were then washed with 0.05% Tween-20, Tris buffered saline (TTBS) and probed with the appropriate secondary antibody in MTBS, goat anti-mouse IgG conjugated with peroxidase (Sigma AP124P, 1:1,000) or goat anti-rabbit IgG conjugated with peroxidase (Invitrogen 32260, 1:2,000). Secondary-probed blots were washed three times with TTBS followed by TBS and incubated with Immobilon chemiluminescent substrate (Millipore). Images were acquired using a ChemiDoc Imager (Bio-Rad) and analyzed using Image Lab software (Bio-Rad). To quantify protein levels, the last image prior to band overexposure was used with MOMP levels serving as the loading control.

### Purification of recombinant proteins

Purification of recombinant GST-tagged RsbW and RsbU or His-tagged RsbV1 and RsbV2 was performed as detailed in reference (39). The 6xHis-3XFLAG-tagged proteins were purified using HisPur cobalt resin (Pierce) as previously described (39). Proteins were induced with 0.3 mM isopropyl β-d-1-thiogalactopyranoside (IPTG) at 18°C for 20 h (His-3XFLAG-RsbV1) or with 0.3 mM IPTG at 30°C for 4 h (His-3XFLAG-RsbV2). Following purification, the 6xHis-tag was cleaved using the WELQut protease (Thermo Scientific) in 100 mM Tris (pH 8.0) at 4°C overnight. The cleaved 6xHis-tag and WELQut protease were removed using HisPur cobalt resin and the supernatants were retained. Preparation of GST-cleaved RsbU and phosphorylated RsbV1 and RsbV2 were performed as detailed in reference (39). Proteins were separated on SDS-PAGE gels to confirm purity and expected product sizes (see Supplemental Material and Fig. S9). *In vitro* protein assays were performed with at least two independently purified protein preparations.

### TEM

HeLa cells were infected at an MOI of 1 with the recombinant *C. trachomatis* L2 strain possessing the plasmid encoding-inducible expression of the FLAG-tagged RsbV2 S55A mutant. At 4 hpi, expression of the construct was induced or not with 10 nM aTc. At 48 hpi, samples were fixed with 2% glutaraldehyde and 2% paraformaldehyde in 0.1 M Sorensen’s phosphate buffer, pH 7.2. At the time of processing, samples were washed with PBS to remove excess fixative, and post fixation was performed for 1 h using a 1% aqueous solution of osmium tetroxide. Samples were dehydrated in a graded ethanol series (50%, 70%, 90%, 95%, and three times of 100% ethanol) with all steps being incubated for 15 min each. Subsequently, samples were soaked in 100% propylene oxide for three times for 15 min each. Samples were kept overnight in the fume hood while soaking in propylene oxide:Embed resin solution (1:1 ratio) until all the propylene oxide was evaporated. The following day, the samples were incubated in fresh resin for 2 h at room temperature, final embedding was performed, and samples polymerized overnight at 65°C. In addition, 90 nm thick sections of the polymerized blocks were cut on a Leica UC7 Ultramicrotome using a Diatome diamond knife. Sections were placed on uncoated 200 mesh copper grids, and staining was performed using 2% uranyl acetate followed by Reynold’s lead citrate. Sections were examined on a transmission electron microscope (Tecnai G2 Spirit TWIN FEI) operated at 80 kV, and images were acquired using an AMT digital imaging system.

### Statistics

GraphPad Prism 6.07 was used to perform all statistical analyses and *P*-values <0.05 were considered significant. Specific statistical tests and replicates are denoted in the respective figure legends.

## SUPPLEMENTAL MATERIAL

Supplemental material spectrum.01846-24-s0001.pdf Fig. S1 to S9; Supplemental Methods.

Supplemental tables spectrum.01846-24-s0002.xlsx Tables S1 to S5.

ASM does not own the copyrights to Supplemental Material that may be linked to, or accessed through, an article. The authors have granted ASM a non-exclusive, world-wide license to publish the Supplemental Material files. Please contact the corresponding author directly for reuse.
