# Supplementary material for: Distinct impacts of each anti-anti-sigma factor ortholog of the chlamydial Rsb partner switching mechanism on development in Chlamydia trachomatis
Source: Microbiol Spectr. 2024 Oct 29;12(12):e01846-24. doi: 10.1128/spectrum.01846-24 (PMC11619594; doi:10.1128/spectrum.01846-24)
Supplement: Supplemental material — Fig. S1 to S9; Supplemental Methods. [file spectrum.01846-24-s0001.pdf]

Supplemental Methods and Data

In vitro kinase and phosphatase assays

RsbW kinase and RsbU phosphatase assays were adapted from our previous studies (1). In brief, 2 µg or 3 µg of the substrate was mixed with enzyme at molar ratios of 1:0.19 (substrate:enzyme) for kinase assays or 1:0.4 (substrate:enzyme) for phosphatase assays. The kinase reaction was initiated by adding 1 mM ATP. Reactions were performed at 30°C and stopped by adding Laemmli buffer with β-mercaptoethanol and heating at 95°C for 5 min. The phosphorylation status of RsbV1/V2 was observed by running samples on SDS-PAGE gels (16% for His-V1/V2) supplemented with 20 µM of Phos binding reagent (APExBIO). Gels were stained with Coomassie brilliant blue and visualized with a Chemidoc Imager.

In vivo phosphorylation status of RsbV1 and RbV2

To assess the phosphorylation status of wild type RsbV2, confluent HeLa monolayers in two 6 well cell culture dishes were infected with *C. trachomatis* L2 434/Bu at an MOI of 5. At 48 hpi, infected cells were collected using glass beads followed by centrifugation at 17,000xg for 10 min at 4°C (all steps). Host cell background was then removed starting by suspension of the pellet with 0.0125% saponin in PBS. Suspended samples were centrifuged at 200xg to separate *Chlamydia* and host cell debris, and the supernatant was diluted in SPG and centrifuged at 17,000xg for 10 min to pellet bacteria. Pellets were then denatured by adding 1 ml of 8 M urea, 0.1% SDS, and 2.5% BME plus 1 µl of DNase I and incubated for 15 min at room temperature. The mixture was passed through a 18G needle with a 3 ml syringe to homogenize the sample and proteins were precipitated by adding 9 ml of 100% ethanol and frozen at -80°C overnight. The solution was then centrifuged at 13,000xg for 30 min and the supernatant was removed. The pellet was resuspended with lysis buffer (10 mM Tris [pH 8.0], 500 mM NaCl, 0.1% Triton-X, 10% glycerol) plus 1 µl of DNase I, and 200 µg rabbit anti-RsbV2 antibody. Protein/antibody binding was allowed to occur at room temperature for 1 hour with end-over-end mixing followed by end-over-end incubation with Pierce Protein A/G magnetic beads for 1 hour at room temperature. Samples were then placed in a magnetic stand and the supernatant was removed. Beads were washed three times in TTBS and once with distilled, deionized water. Beads were then mixed with Laemmli buffer with β-mercaptoethanol, and incubated at room temperature for 10 minutes with mixing.

For phosphorylation analysis of N-terminal 3xFLAG-tagged RsbV1 or C-terminal 3xFLAG-tagged RsbV2, recombinant *C. trachomatis* harboring plasmid p2TK2Spec-SW2 mCh(GroL2) Tet-3xFLAG-*rsbV1* or p2TK2Spec-SW2 mCh(GroL2) Tet-*rsbV2*-3xFLAG were used to infect HeLa cells grown in 6 well dishes at an MOI of 0.5. Protein production was induced by adding 10 nM aTc at 4 hpi. Cell lysates were harvested at different time points (20, 22, 24, 36, 48 hpi for 3xFLAG RsbV1 and 24, 36, 48 hpi for RsbV2 3xFLAG) by adding Laemmli buffer with β-mercaptoethanol and heating at 95°C

for 5 min. For detection of 3xFLAG RsbV1 at 15 hpi, HeLa cells were grown in a T75 flask and infected at an MOI of 5. Host cell background for the T75 samples was removed as mentioned previously for the 6 well format except that detached cells were centrifuged at 13,000xg instead of 17,000xg and cell pellets were directly lysed in Laemmli buffer with β-mercaptoethanol. For detecting RsbV2 3xFLAG phosphorylation status under different glucose levels, HeLa cells were grown in either high glucose (4.5 g/L) or low glucose (1 g/L) DMEM (adapted to low glucose as described in (1)) in two 6 well plates and infected at an MOI of 0.5. Protein was induced with 10 nM aTc at 4 hpi, and infected cells were harvested at 48 hpi followed by the host cell minimum background procedure. The bacterial pellet was lysed with Laemmli buffer and heated at 95°C for 5 min. As a phosphorylation negative control, *C. trachomatis* harboring the plasmid p2TK2Spec-SW2 mCh(GroL2) Tet-*rsbV2* S55A-3xFLAG was used in the HeLa cell infection assays.

Protein phosphorylation was confirmed by running samples on 16% SDS-PAGE gels supplemented with Phos binding reagent (20 µM for 3xFLAG-RsbV1 and RsbV2-3xFLAG or 50 µM for RsbV2). After electrophoresis, the Phos binding reagent was chelated in the gels with 10 mM EDTA in western blot transfer buffer with gentle agitation for 10 minutes with three buffer exchanges. The gel was then washed with transfer buffer without EDTA for 10 minutes, three times. Protein was transferred to nitrocellulose membrane at 89V for 2 h. RsbV2 and 3xFLAG were detected as described in the main text.

RsbV1/V2/RsbW *E. coli* co-expression

An *E. coli* surrogate *in vivo* co-expression model was used to assess the impact of mutations and/or tag-locations on the phosphorylation of RsbV1 and RsbV2 by RsbW. The *rsbW* gene was cloned into the pACYCDuet^{TM}-1 expression vector (chloramphenicol resistant, IPTG-inducible) using standard restriction digestion cloning methods to generate an N-terminal 6xHis-tagged RsbW. FLAG-tagged *rsbV1* and *rsbV2* were delivered via p2TK2Spec-SW2 mCh(GroL2) Tet (spectinomycin resistant, aTc-inducible) and wild-type *rsbV2* or *rsbV2* S55A were provided via pLATE11 (ampicillin resistant, IPTG-inducible, LIC-cloned as directed by the manufacturer). Dual transformant (*rsbW* plus *rsbV1* or *rsbv2*) BL21(de3) strains were grown at 37°C with the required antibiotics until reaching an OD_{600nm} of 0.6 to 0.8 and induced for 5 hours with 1 mM IPTG and 50 ng/ml aTc (pACYC and p2TK2 vectors) or induced for 2.5 hours with 1 mM IPTG (pACYC and pLATE11 vectors). Post-induction samples were taken and lysed with Laemmli buffer with β-mercaptoethanol, heated at 95°C, and resolved on 15% SDS-PAGE gels for protein detection via western blot or on 16% Phos-tag SDS-PAGE gels (20 µM Phos binding reagent for 3xFLAG-RsbV1 and RsbV2-3xFLAG or 50 µM for RsbV2) followed by western blot to assess RsbV1/RsbV2 phosphorylation. Western blotting was performed as described in-text for FLAG-tag or RsbV2 detection.

Table S1. Percent Identity of AASFs.

Table S2. Bacterial abbreviations and NCBI IDs for Figure 2/Figure S1.

Table S3. Bacterial strains used in this study.

Table S4. Primers used in this study.

Table S5. gBlocks used in this study.

Figure S1. Amino acid sequence alignment of RsbV from representative species. AASF amino acid sequences were selected from Chlamydiales (RsbV1, dark blue; RsbV2, sky blue) Amoebachlamydiales, Anoxychlamydiales, Simkaniales (nightingale-brown), and other species (gray) for alignment using MUSCLE. The multiple alignment sequence was visualized using Bioedit. A 40% threshold was used to identify conserved residues with identical residues in black and similar residues in gray. Secondary structure (α-folds as green and β-sheets as purple) was annotated based on the AlphaFold predicted RsbV2 protein structure from *C. trachomatis* L2 434/Bu. The red arrow denotes the phosphorylation site serine based on prior data for RsbV1 and RsbV2 in *C. trachomatis* (2) and *C. caviae* (3). The SpoIIAB (RsbW homolog) interaction site with SpoIIAA (RsbV homolog) is marked with pink arrows (4) or orange arrows (5). NCBI IDs and species abbreviations are listed in Table S2.

Figure S2. Structural modeling of the RsbV2/RsbW and RsbV1/RsbW interaction sites. Protein structures of RsbV1, RsbV2, and RsbW were predicted using AlphaFold. The predicted structures were superimposed on the crystal structure of SpoIIAA-SpoIIAB (5), rendered using UCSF Chimera, and the template was removed. The bottom panel shows an RsbW dimer binding to RsbV1 or RsbV2. In the center, RsbV1-RsbW or RsbV2-RsbW structural faces are shown on same side. The side panels (side interactions between V1[left]/V2[right] and W) and top panels (bottom interactions between V1[top left]/V2[top right] and W top) show the surface hydrophobicity profiles for RsbV1 and RsbV2.

Figure S3. Assessment of pH impact on phosphorylation and dephosphorylation of RsbV1 and RsbV2. RsbW kinase assays (A) and RsbU phosphatase assays (B) were assessed at pH 7.28 (predicted chlamydial cytoplasmic pH, (6)) or pH 7.5 (prior studies, (1)). Kinase and phosphatase enzyme activities were assessed over time towards RsbV1 (left panels) or RsbV2 (right panels). The phosphorylation status of AASFs was analyzed by running samples on 16% Phos-tag SDS-PAGE gels and proteins were detected with Coomassie Brilliant Blue staining. The location of phosphorylated and non-phosphorylated species are listed to the left (RsbV1) or right (RsbV2) of the gels.

Figure S4. Phosphorylation/dephosphorylation of RsbV1/RsbV2 with 3X FLAG tags at the N- or C-terminus. Phosphorylation status of N-terminal 3xFLAG-tagged RsbV1 (A) and C-terminal 3XFLAG-tagged RsbV2 (B) were assessed by running samples on 16% Phos-tag SDS-PAGE gels followed by western blot with an anti-FLAG antibody (n≥2). A) N-term 3xFLAG RsbV1 (labeled as 3XF-V1) was induced at 4 hpi and the samples were harvested at 15, 20, 22, 24, 36, and 48 hpi. Except for the 15 hpi trial,

cells were infected at an MOI of 0.5 in a 6 well plate. For 15 hpi, cells were infected at an MOI of 5 in a T75 flask. Host cell background was removed before lysis in Laemmli buffer. Expected positions of non-phosphorylated and phosphorylated proteins are listed to the left and right of gels. B) C-ter 3xFLAG-tagged RsbV2 (labeled as V2-3XF) was induced in infected cells cultured in either high (HG) or low (LG) glucose DMEM and harvested at 48 hpi (left) or at 24, 36, and 48 hpi in high glucose DMEM. For protein running controls of phosphorylated/non-phosphorylated species, RsbV1 or RsbV2 were co-induced with RsbW in *E.coli* (A and B). C) and D) Kinase and phosphatase assays were performed using affinity purified His-tagged (labeled as H) and/or FLAG-tagged (labeled as 3F) proteins to test the impact of the 3xFLAG tag location on RsbW and RsbU activity towards RsbV1/V2. Reactions were performed with or without ATP using an overnight incubation (kinase assay in [D]) or by incubating for 0 minutes or overnight (kinase [C], phosphatase [D] assays). Phosphorylation status was confirmed on 16% Phos-tag SDS-PAGE gels. Protein was detected using Coomassie Brilliant Blue staining (n≥2).

Figure S5. Confirmation of induction of RsbV1 S56A and RsbV2 S55A by immunofluorescence microscopy or western blot. Recombinant *C. trachomatis* L2 producing FLAG-tagged RsbV1 S56A or RsbV2 S55A were used to infect HeLa cells. A) N-terminal tagged proteins were induced by adding 10 nM aTc at 4 hpi, and samples were harvested at 48 hpi. Western blot using an anti-FLAG antibody confirmed protein induction. B) RsbV1 S56A or RsbV2 S55A were induced by adding 112 nM aTc at 4 hpi and cells were fixed at 48 hpi as described in the main text. Detection was performed with anti-FLAG antibody (1:500) followed by a secondary antibody conjugated with Alexa Fluor 488 (1:2000). Anti-FLAG and mCherry (recombinant vector-encoded) images were taken at 1000x, and the brightness was adjusted in ImageJ; scale bars are 20 µm.

Figure S6. Representative immunofluorescence images used to determine the impact of RsbV2 S55A expression on inclusion size and confirmation of induced recombinant protein. C-terminal FLAG-tagged wild type or S55A mutant RsbV2 was induced by adding 10 nM aTc at 4 hpi. (A) Cells were fixed at 48 hpi and immunofluorescence microscopy was performed with an anti-MOMP antibody and an anti-mouse secondary conjugated with Alexa Fluor 488. Images were acquired at 1000x and data were acquired for MOMP (green), vector-encoded mCherry (magenta), and DNA via DAPI staining (blue). Scale bars are 20 µm. Images were processed using ImageJ and MOMP staining was used to measure inclusion size, reported in Figure 5 (F) for 36 hpi and Figure S6 (B) for 24 hpi and 48 hpi. Statistical analysis was done by t-test (ns = $P > 0.05$, ** = $P \le 0.01$, **** = $P \le 0.0001$). C) Western blot with anti-MOMP, anti-FLAG, or anti-RsbV2 antibodies was performed on similarly infected cells as (A) to confirm induction of recombinant RsbV2 protein. Induction samples of RsbV2-3xFLAG/RsbV2 S55A 3xFLAG from *E.coli* were used as a positive control (+).

Figure S7. Expression of a His-tagged RsbV2 S55A mutant reduces infectious progeny production. N-terminal His-tagged recombinant RsbV2 S55A was expressed in *C. trachomatis* L2. Infectious progenies were measured using the IFU assay. A) Cells

were infected at an MOI of 0.5. RsbV2 S55A production was induced at different time points (0, 4, 8, 12, 16, or 20 hpi), and samples were harvested at 48 hpi (n≥2). Statistical analysis was done by t-test comparing results to the 4 hpi induction time point. B) Cells were infected at different MOIs (0.5, 1, or 5), and RsbV2 S55A was induced at 4 hpi. Samples were harvested at 24 or 48 hpi and titered by IFU. (n=2) C) Cells were infected at an MOI of 0.5 and RsbV2 S55A was induced at 4 hpi. The samples were harvested at 24, 30, 36, 60, or 72 hpi for titering by IFU assay (n=2). Statistical analysis was done by t-test (ns = $P > 0.05$, * = $P \leq 0.05$).

Figure S8. TEM images of infected cells under conditions with uninduced or induced RsbV2 S55A. Recombinant *C. trachomatis* L2 producing FLAG-tagged RsbV2 S55A was used to infect HeLa cells. RsbV2 was induced by adding 10 nM aTc and samples were processed for TEM at 48 hpi. Left images represent uninduced samples, and right images are induced samples. Magnification and scale bar sizes are listed to the left of the panels. Arrows show representative normal EBs (black), normal IBs (gray), an abnormal IB (brown), or RBs (white). Images outlined in purple (uninduced) or dark blue (induced) are from the same respective inclusions.

Figure S9. Protein preparations used in this study that were not previously reported. 1 or 5 µg of affinity purified proteins were loaded on 15% SDS-PAGE gels. 3FV1 (3XF-RsbV1) or 3FV2 (3XF-RsbV2) was prepared by cleaving the His-tag from His3FV2 (His-3XF-RsbV1) or His3FV1 (His-3XF-RsbV2). Protein types are listed at the top of each gel, molecular weight markers are listed to the left of gels. Expected proteins sizes: His-RsbV2-3XF (18.31kDa), His3FV1 (18.35 kDa), His3FV2 (18.23 kDa), 3FV1 (15.18 kDa), 3FV2 (15.06 kDa). All proteins were detected by Coomassie Brilliant Blue staining.

References

1. Kuwabara S, Landers ER, Fisher DJ. 2022. Impact of nutrients on the function of the chlamydial Rsb partner switching mechanism. Pathog Dis 80.
2. Hua L, Hefty PS, Lee YJ, Lee YM, Stephens RS, Price CW. 2006. Core of the partner switching signalling mechanism is conserved in the obligate intracellular pathogen *Chlamydia trachomatis*. Mol Microbiol 59:623-36.
3. Fisher DJ, Adams NE, Maurelli AT. 2015. Phosphoproteomic analysis of the *Chlamydia caviae* elementary body and reticulate body forms. Microbiology (Reading) 161:1648-1658.
4. Pathak D, Jin KS, Tandukar S, Kim JH, Kwon E, Kim DY. 2020. Structural insights into the regulation of SigB activity by RsbV and RsbW. Iucrj 7:737-747.
5. Masuda S, Murakami KS, Wang S, Anders Olson C, Donigian J, Leon F, Darst SA, Campbell EA. 2004. Crystal structures of the ADP and ATP bound forms of the *Bacillus* anti-sigma factor SpoIIAB in complex with the anti-anti-sigma SpoIIAA. J Mol Biol 340:941-56.
6. Grieshaber S, Swanson JA, Hackstadt T. 2002. Determination of the physical environment within the inclusion using ion-selective ratiometric probes. Cellular Microbiology 4:273-283.

7. Shen L, Gao L, Swoboda AR, Ouellette SP. 2024. Targeted repression of *topA* by CRISPRi reveals a critical function for balanced DNA topoisomerase I activity in the *Chlamydia trachomatis* developmental cycle. mBio 15.
8. Lowden NM, Yeruva L, Johnson CM, Bowlin AK, Fisher DJ. 2015. Use of aminoglycoside 3' adenyltransferase as a selection marker for *Chlamydia trachomatis* intron-mutagenesis and *in vivo* intron stability. BMC Res Notes 8:570.

Figure S1

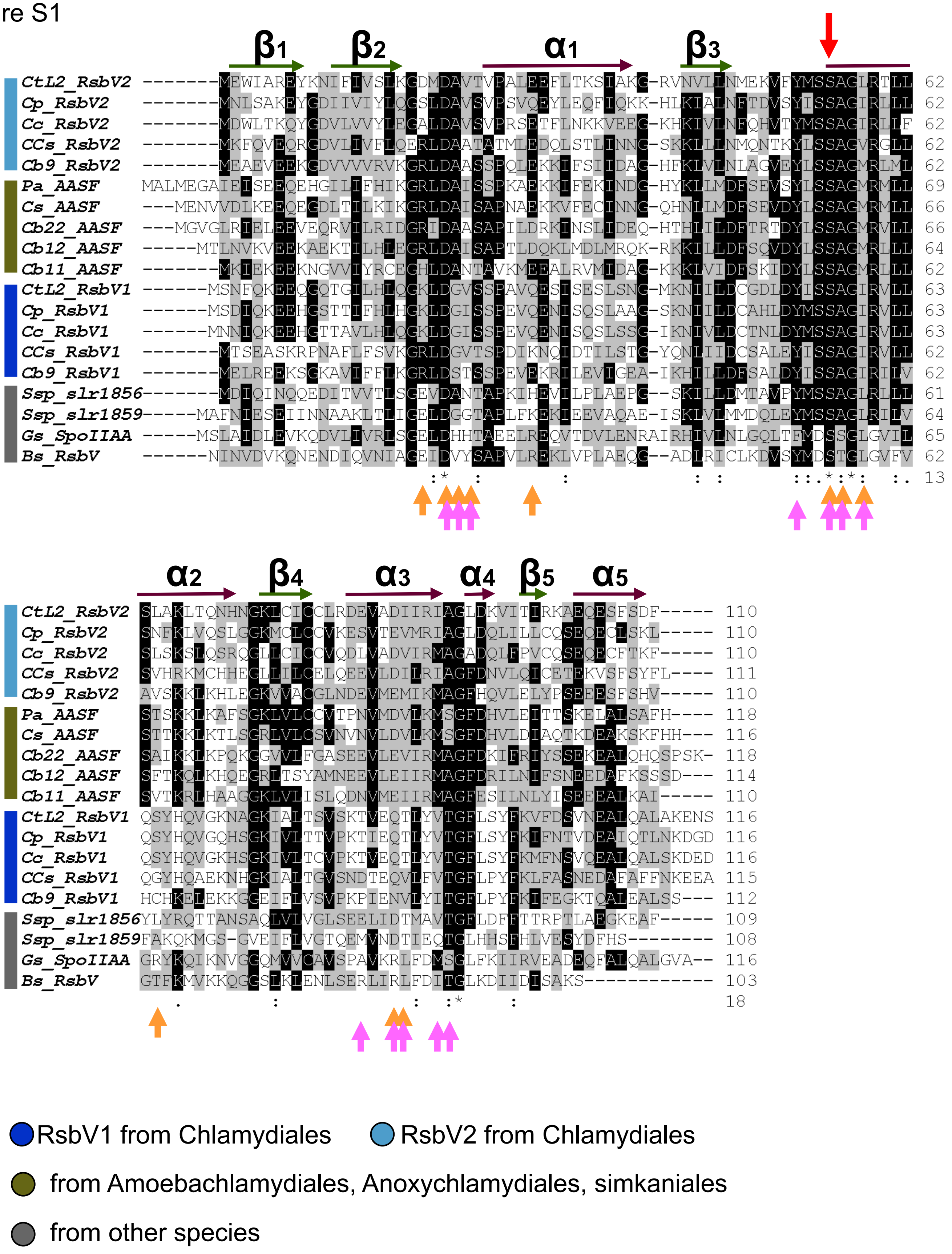
β1 β2 α1 β3
CtL2_RsbV2 -------MEWIAREYKNIFIVSLKGDMDAVTVPALEEFLTKSIAKG-RVNVLLNMEKVFYMSSAGLRTLL 62
Cp_RsbV2 -------MNLSAKEYGDIIVIYLQGSLDAVSVPSVQEYLEQFIQKK-HLKIALNFTDVSYISSAGIRLLL 62
Cc_RsbV2 -------MDWLTKQYGDVLVVYLEGALDAVSVPRSETFLNKKVEEG-KHKIVLNFQHVTYMSSAGIRLIF 62
CCs_RsbV2 -------MKFQVEQRGDVLIVFLQERLDAATATMLEDQLSTLINNG-SKKLLLNMQNTKYLSSAGVRGLL 62
Cb9_RsbV2 -------MEAEVEEKGDVVVVRVKGRLDAASSPQLEKKIFSIIDAG-HFKLVLNLAGVEYLSSAGMRLML 62
Pa_AASF MALMEGAIEISEEQEHGILIFHIKGRLDAISSPKAEKKIFEKINDG-HYKLLMDFSEVSYLSSAGMRMLL 69
Cs_AASF ---MENVVDLKEEQEGDLTILKIKGRLDAISAPNAEKKVFECINNG-QHNLLMDFSEVDYLSSAGMRMLL 66
Cb22_AASF ---MGVGLRIELEEVEQRVILRIDGRIDAASAPILDRKINSLIDEQ-HTHLILDFTRTDYLSSAGMRVLL 66
Cb12_AASF -----MTLNVKVEEKAEKTILHLEGRLDAISAPTLDQKLMDLMRQK-RKKILLDFSQVDYLSSAGMRLLL 64
Cb11_AASF -------MKIEKEEKNGVVIYRCEGHLDANTAVKMEEALRVMIDAG-KKKLVIDFSKIDYLSSAGMRLLL 62
CtL2_RsbV1 ------MSNFQKEEQGQTGILHLQGKLDGVSSPAVQESISESLSNG-MKNIILDCGDLDYISSAGIRVLL 63
Cp_RsbV1 ------MSDIQKEEHGSTTIFHLHGKLDGISSPEVQENISQSLAAG-SKNIILDCAHLDYMSSAGIRVLL 63
Cc_RsbV1 ------MNNIQKEEHGTTAVLHLQGKLDGISSPEVQENISQSLSSG-IKNIVLDCTNLDYMSSAGIRVLL 63
CCs_RsbV1 -------MTSEASKRPNAFLFSVKGRLDGVTSPDIKNQIDTILSTG-YQNLIIDCSALEYISSAGIRVLL 62
Cb9_RsbV1 -------MELREEKSGKAVIFFLKGRLDSTSSPEVEKRILEVIGEA-IKHILLDFSALDYISSAGIRILV 62
Ssp_slr1856-------MDIQINQQEDITVVTLSGEVDANTAPKIHEVILPLAEPG--SKILLDMTAVPYMSSAGLRLLL 61
Ssp_slr1859-----MAFNIESEIINNAAKLTLIGELDGGTAPLFKEKIEEVAQAE-ISKLVLMMDQLEYMSSAGLRILV 64
Gs_SpoIIAA -----MSLAIDLEVKQDVLIVRLSGELDHHTAEELREQVTDVLENRAIRHIVLNLGQLTFMDSSGLGVIL 65
Bs_RsbV ------NINVDVKQNENDIQVNIAGEIDVYSAPVLREKLVPLAEQG--ADLRICLKDVSYMDSTGLGVFV 62
:* : : : : ::.*:*: :. 13
α2 β4 α3 α4 β5 α5
CtL2_RsbV2 SLAKLTQNHNGKLCICCLRDEVADIIRIAGLDKVITIRKAEQESFSDF----- 110
Cp_RsbV2 SNFKLVQSLGGKMCLCCVKESVTEVMRIAGLDQLILLCQSEQECLSKL----- 110
Cc_RsbV2 SLSKSLQSRQGLLCICCVQDLVADVIRMAGADQLFPVCQSEQECFTKF----- 110
CCs_RsbV2 SVHRKMCHHEGLLILCELQEEVLDILRIAGFDNVLQICETEKVSFSYFL---- 111
Cb9_RsbV2 AVSKKLKHLEGKVVACGLNDEVMEMIKMAGFHQVLELYPSEEESFSHV----- 110
Pa_AASF STSKKLKAFSGKLVLCCVTPNVMDVLKMSGFDHVLEITTSKELALSAFH---- 118
Cs_AASF STTKKLKTLSGRLVLCSVNVNVLDVLKMSGFDHVLDIAQTKDEAKSKFHH--- 116
Cb22_AASF SAIKKLKPQKGGVVLFGASEEVLEVIRMAGFDKIFRIYSSEKEALQHQSPSK- 118
Cb12_AASF SFTKQLKHQEGRLTSYAMNEEVLEIIRMAGFDRILNIFSNEEDAFKSSSD--- 114
Cb11_AASF SVTKRLHAAGGKLVLISLQDNVMEIIRMAGFESILNLYISEEEALKAI----- 110
CtL2_RsbV1 QSYHQVGKNAGKIALTSVSKTVEQTLYVTGFLSYFKVFDSVNEALQALAKENS 116
Cp_RsbV1 QSYHQVGQHSGKIVLTTVPKTIEQTLYVTGFLSYFKIFNTVDEAIQTLNKDGD 116
Cc_RsbV1 QSYHQVGKHSGKIVLTCVPKTVEQTLYVTGFLSYFKMFNSVQEALQALSKDED 116
CCs_RsbV1 QGYHQAEKNHGKIALTGVSNDTEQVLFVTGFLPYFKLFASNEDAFAFFNKEEA 115
Cb9_RsbV1 HCHKELEKKGGEIFLVSVPKPIENVLYITGFLPYFKIFEGKTQALEALSS--- 112
Ssp_slr1856YLYRQTTANSAQLVLVGLSEELIDTMAVTGFLDFFTTRPTLAEGKEAF----- 109
Ssp_slr1859FAKQKMGS-GVEIFLVGTQEMVNDTIEQTGLHHSFHLVESYDFHS-------- 108
Gs_SpoIIAA GRYKQIKNVGGQMVVCAVSPAVKRLFDMSGLFKIIRVEADEQFALQALGVA-- 116
Bs_RsbV GTFKMVKKQGGSLKLENLSERLIRLFDITGLKDIIDISAKS------------ 103
. : : :* : 18
RsbV1 from Chlamydiales
RsbV2 from Chlamydiales
from Amoebachlamydiales, Anoxychlamydiales, simkaniales
from other species

Figure S2

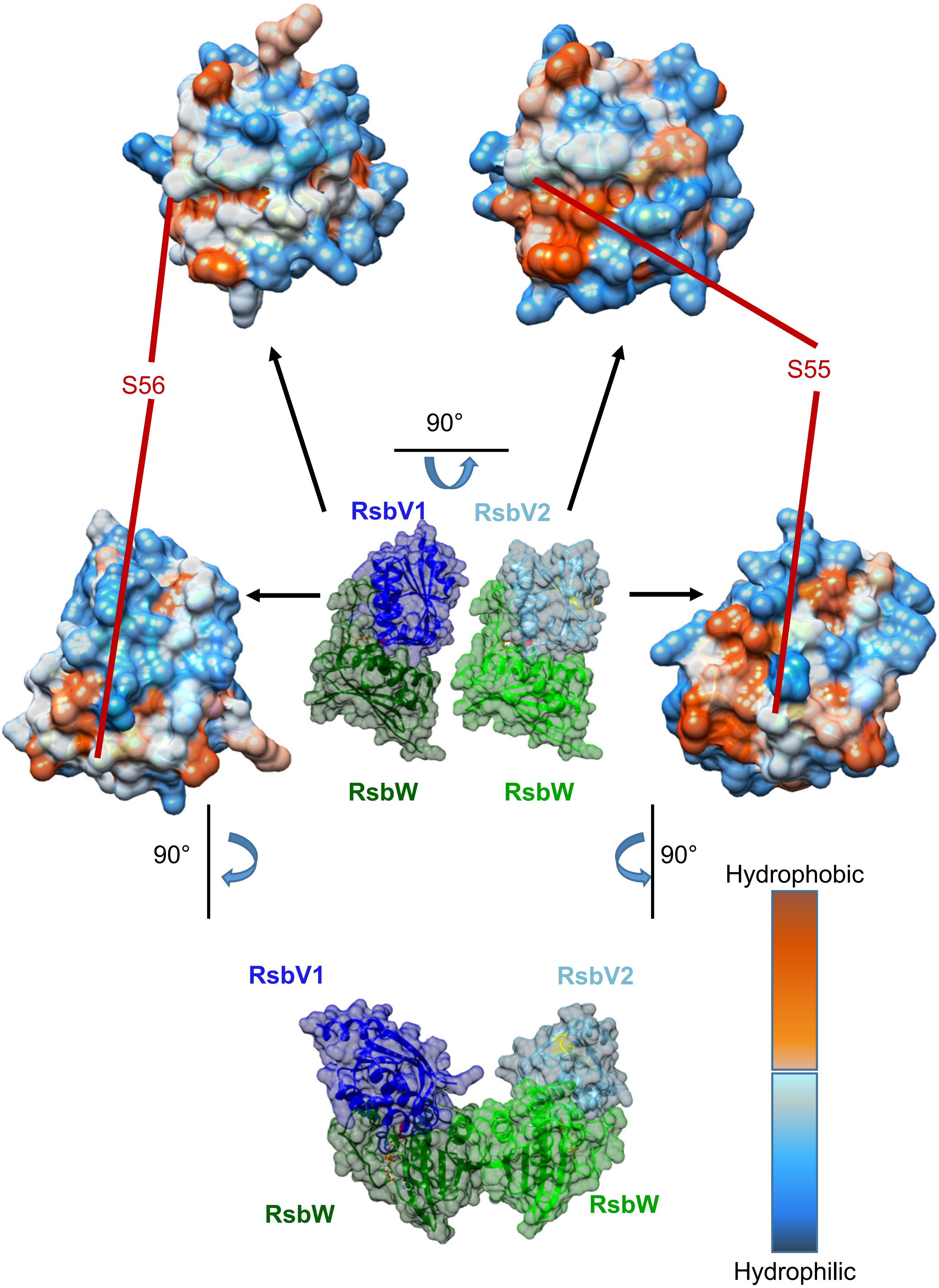
S56
S55
90°
RsbV1
RsbV2
RsbW
RsbW
90°
90°
RsbV1
RsbV2
RsbW
RsbW
Hydrophobic
Hydrophilic

Figure S3

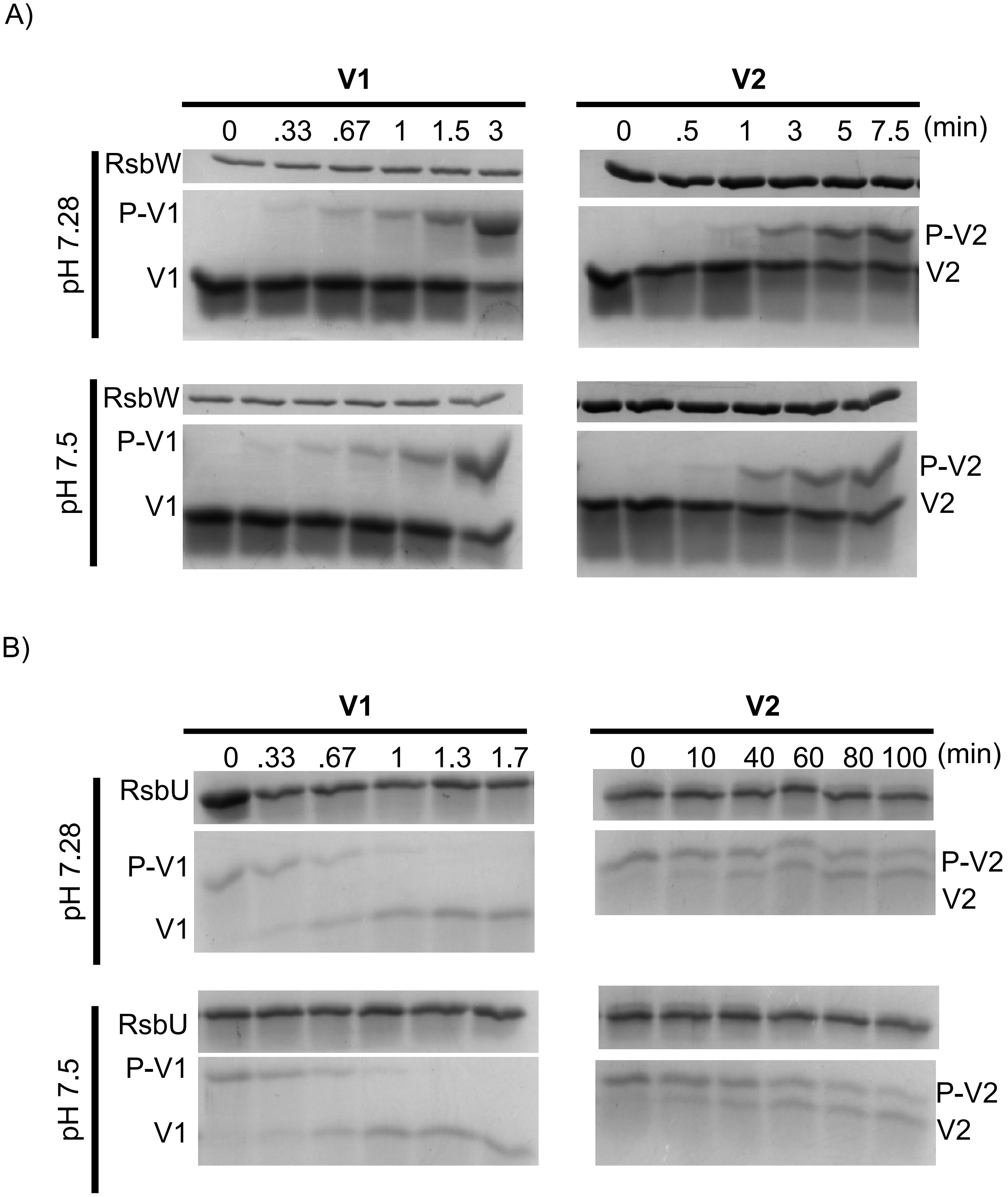
A)
V1
V2
0 .33 .67 1 1.5 3
0 .5 1 3 5 7.5 (min)
RsbW
P-V1
V1
P-V2
V2
pH 7.28
pH 7.5
B)
V1
V2
0 .33 .67 1 1.3 1.7
0 10 40 60 80 100 (min)
RsbU
P-V1
V1
P-V2
V2
pH 7.28
pH 7.5

Figure S4

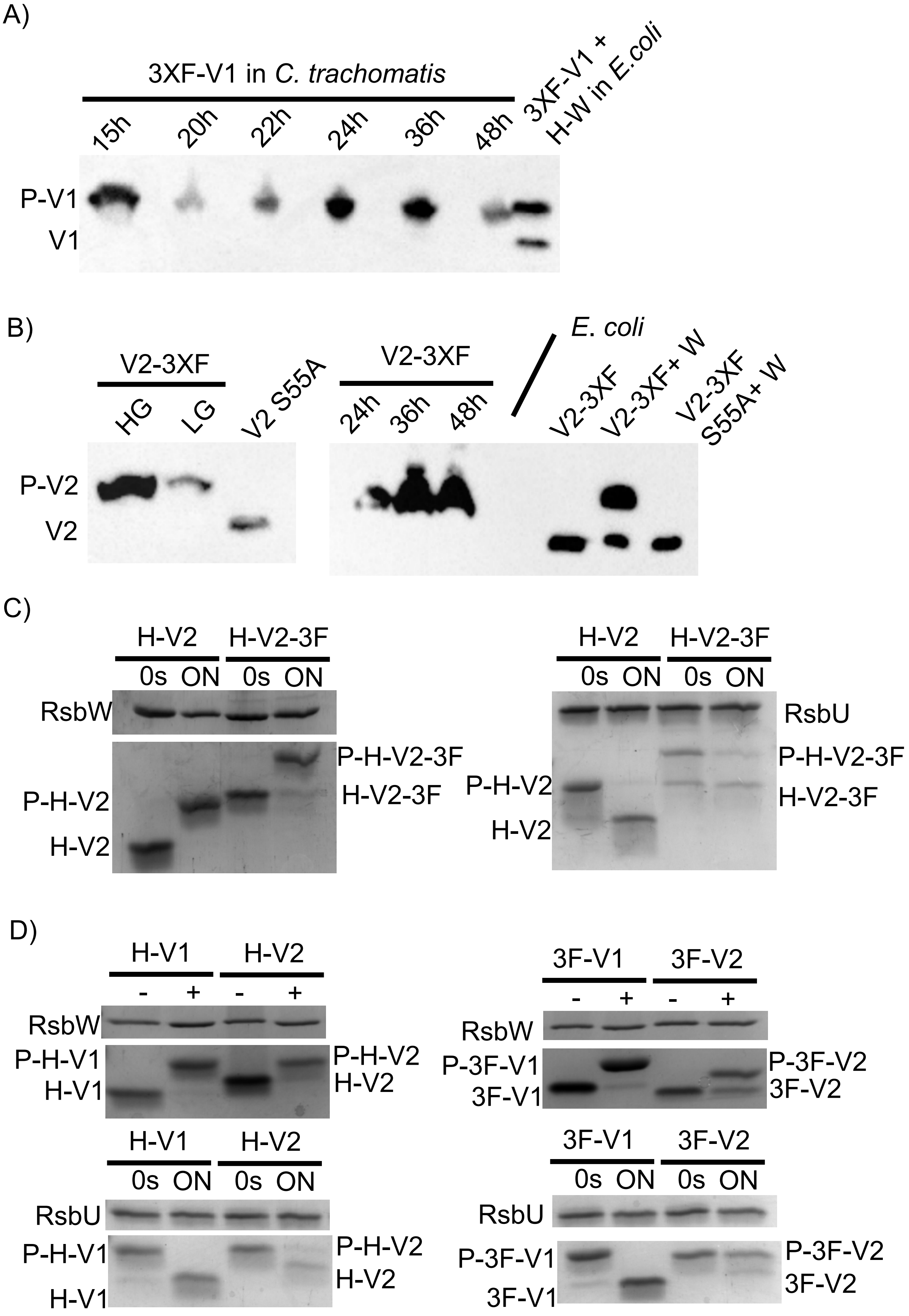

A)
3XF-V1 in *C. trachomatis*
15h
20h
22h
24h
36h
48h
3XF-V1 + H-W in *E.coli*
P-V1
V1
B)
V2-3XF
HG
LG
V2 S55A
V2-3XF
24h
36h
48h
E. coli
V2-3XF
V2-3XF+ W
V2-3XF S55A+ W
P-V2
V2
C)
H-V2
H-V2-3F
0s
ON
0s
ON
RsbW
P-H-V2-3F
H-V2-3F
P-H-V2
H-V2
H-V2
H-V2-3F
0s
ON
0s
ON
RsbU
P-H-V2-3F
H-V2-3F
P-H-V2
H-V2
D)
H-V1
H-V2
-
+
-
+
RsbW
P-H-V1
H-V1
P-H-V2
H-V2
3F-V1
3F-V2
-
+
-
+
RsbW
P-3F-V1
3F-V1
P-3F-V2
3F-V2
H-V1
H-V2
0s
ON
0s
ON
RsbU
P-H-V1
H-V1
P-H-V2
H-V2
3F-V1
3F-V2
0s
ON
0s
ON
RsbU
P-3F-V1
3F-V1
P-3F-V2
3F-V2

Figure S5

A) N-terminal 3XFLAG-tagged constructs

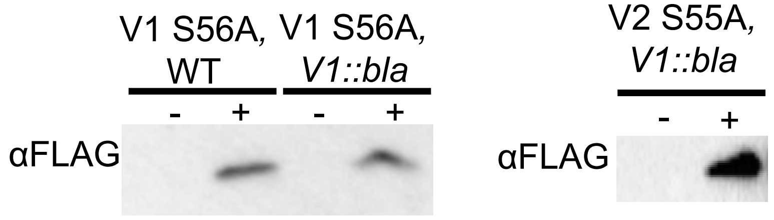

B)

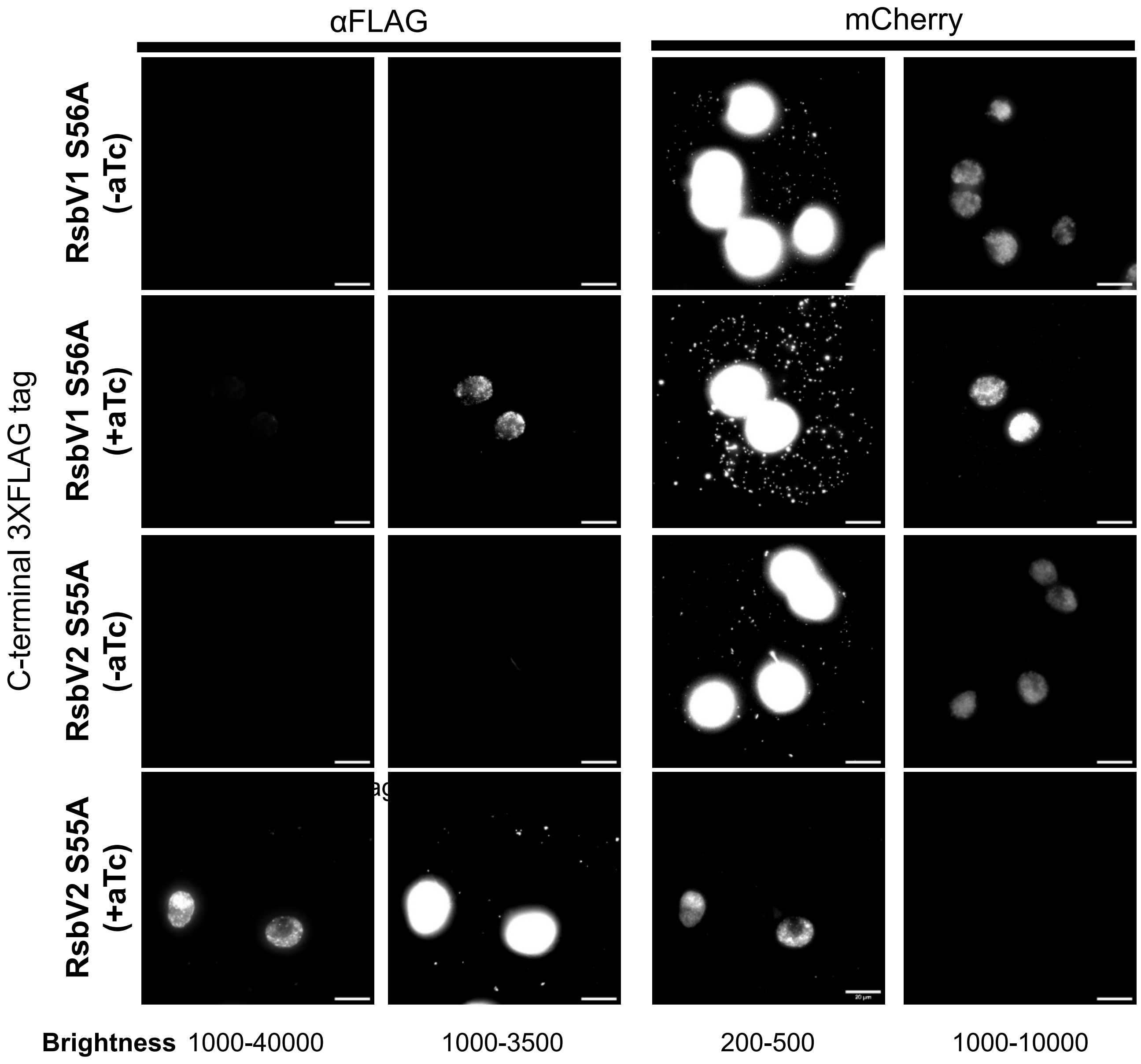

Figure S6

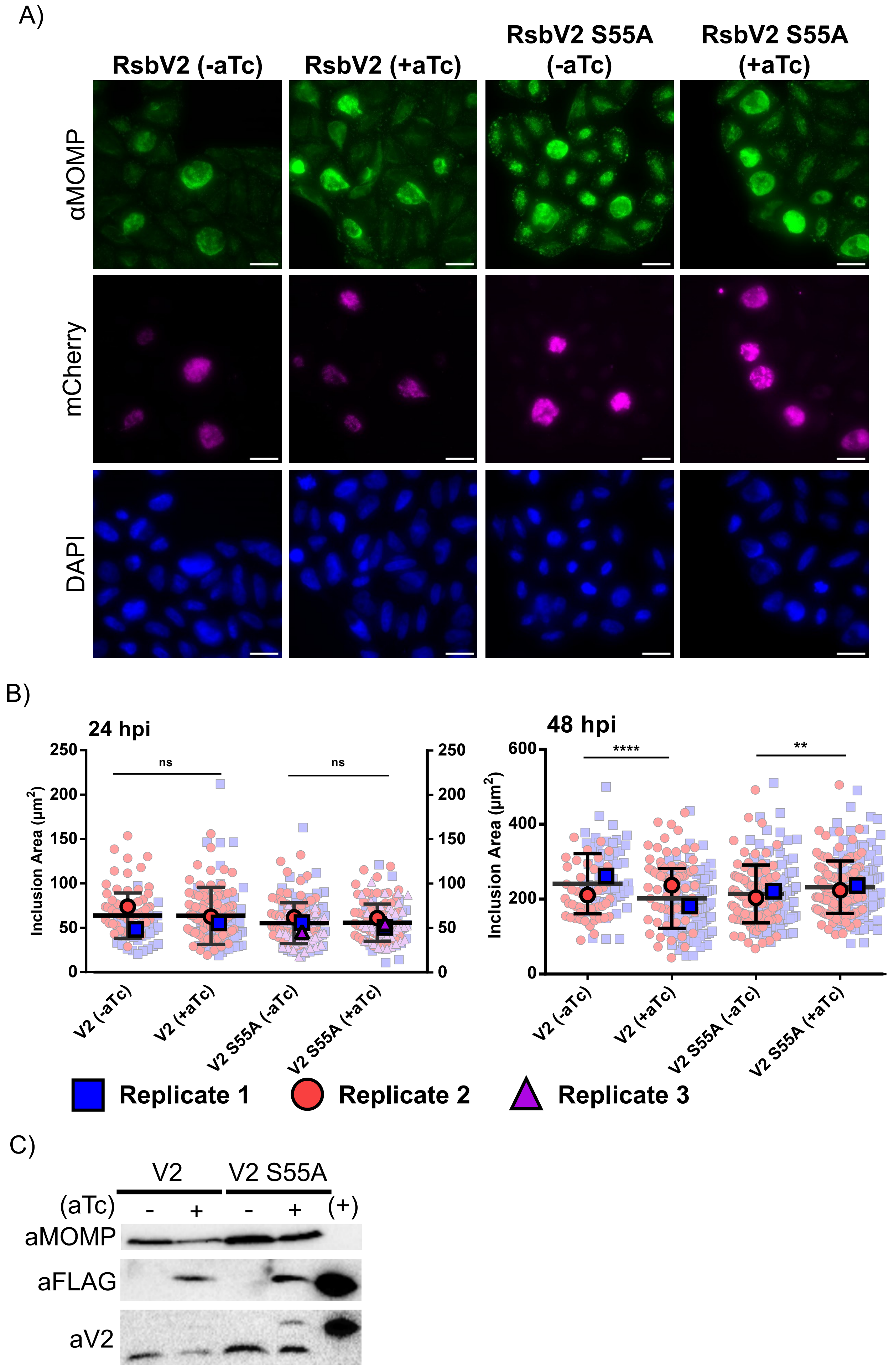
A)
RsbV2 (-aTc)
RsbV2 (+aTc)
RsbV2 S55A (-aTc)
RsbV2 S55A (+aTc)
αMOMP
mCherry
DAPI
B)
24 hpi
48 hpi
Inclusion Area (μm²)
ns
****
**
V2 (-aTc)
V2 (+aTc)
V2 S55A (-aTc)
V2 S55A (+aTc)
Replicate 1
Replicate 2
Replicate 3
C)
V2
V2 S55A
(aTc)
- + - + (+)
aMOMP
aFLAG
aV2

Figure S7

A)

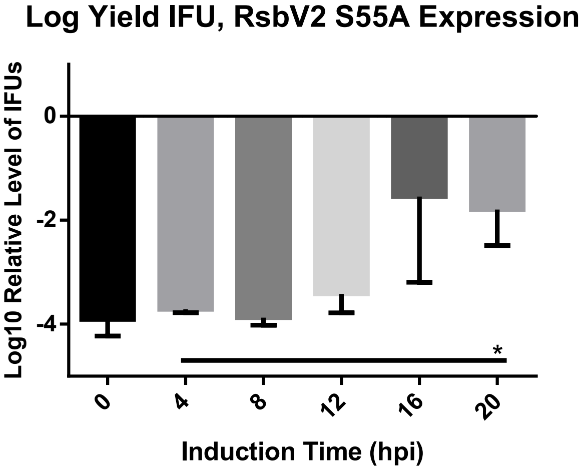

B)

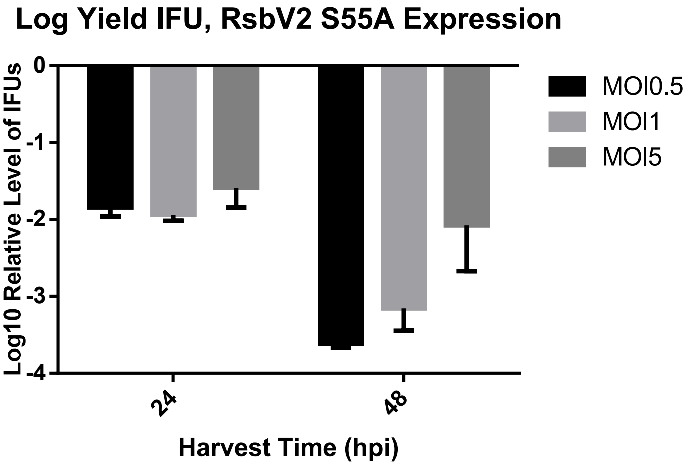

C)

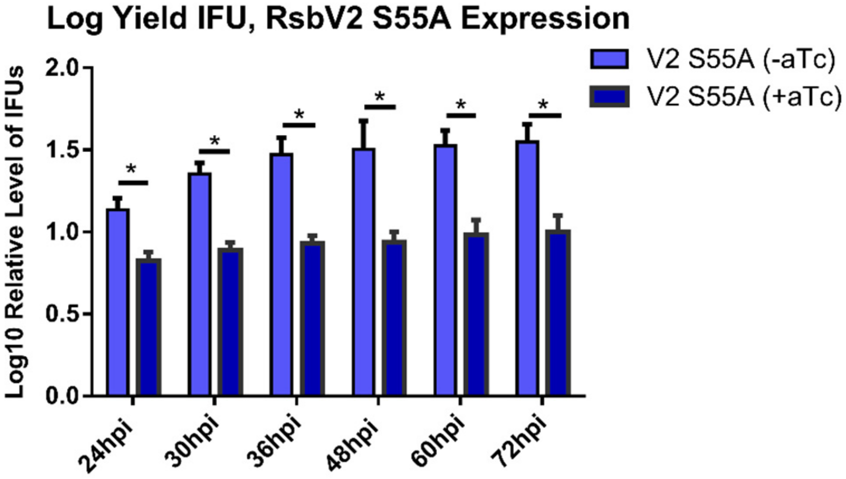

Figure S8

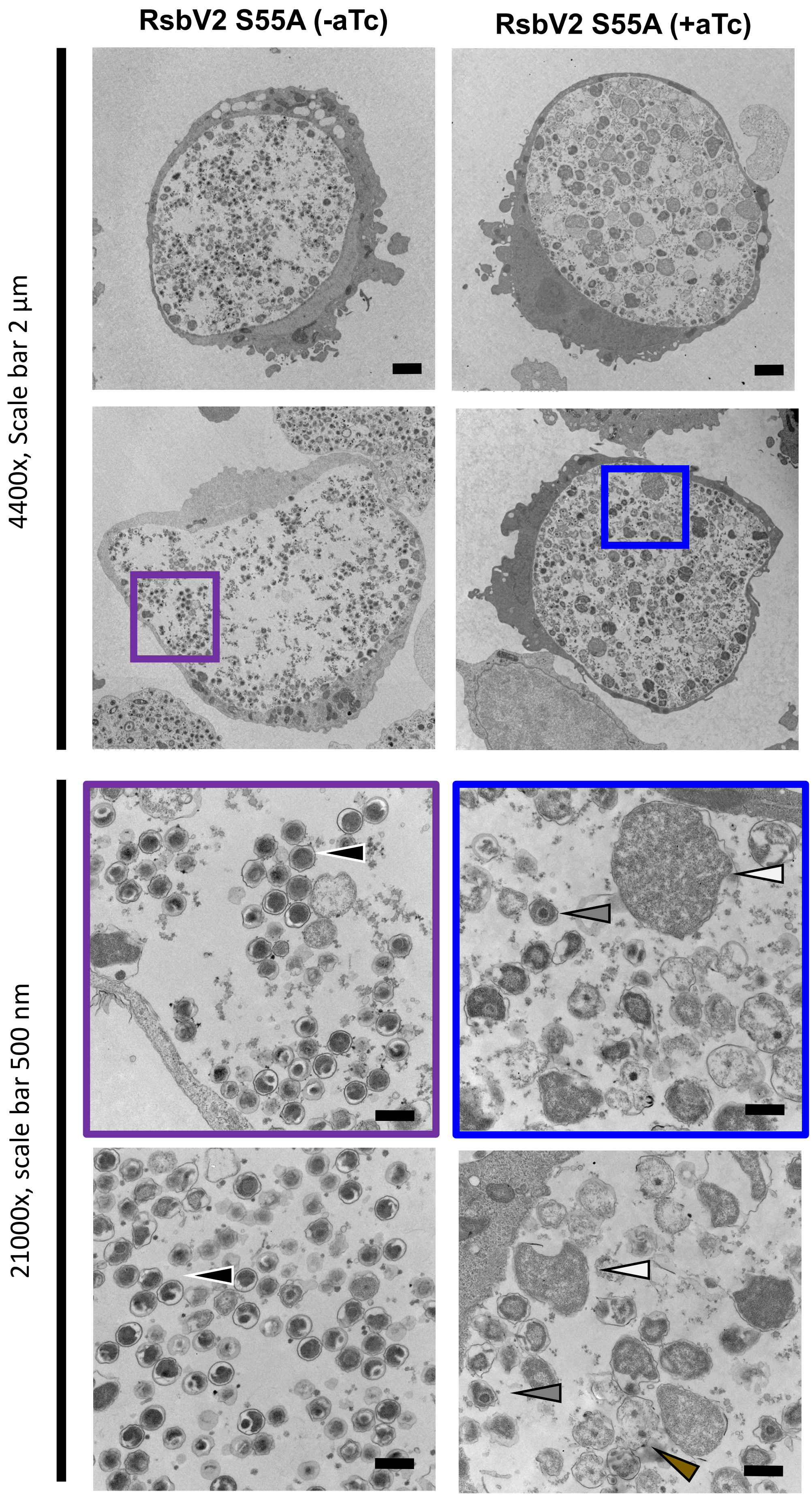

RsbV2 S55A (-aTc)
RsbV2 S55A (+aTc)
4400x, Scale bar 2 µm
21000x, scale bar 500 nm

Figure S9

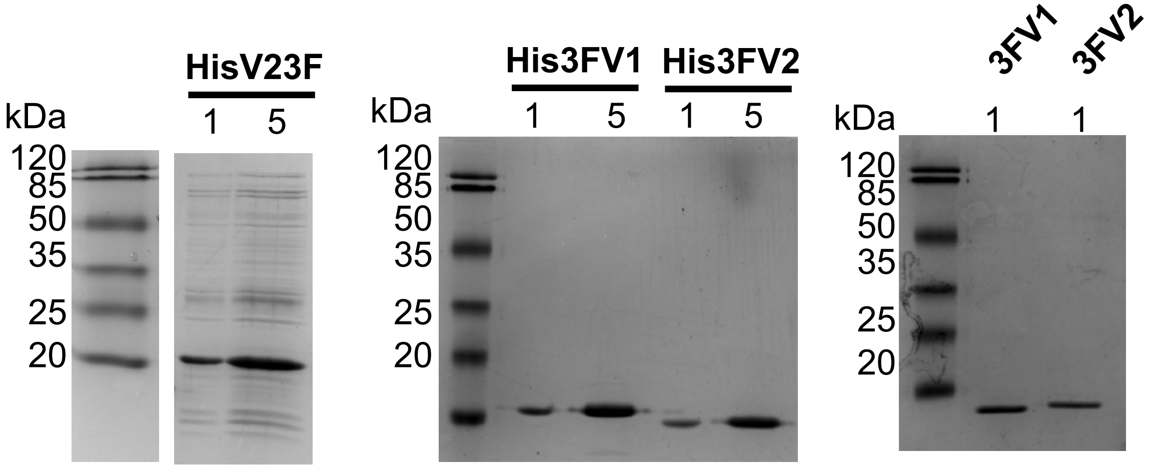

HisV23F
kDa
1
5
120
85
50
35
25
20
His3FV1
His3FV2
kDa
1
5
1
5
120
85
50
35
25
20
3FV1
3FV2
kDa
1
1
120
85
50
35
25
20
